# Divergent patterns of zooplankton connectivity in the epipelagic and mesopelagic zones of the eastern North Pacific

**DOI:** 10.1002/ece3.10664

**Published:** 2023-11-05

**Authors:** Stephanie A. Matthews, Leocadio Blanco‐Bercial

**Affiliations:** ^1^ California Current Ecosystem Long‐Term Ecological Research Site, Integrative Oceanography Division, Scripps Institution of Oceanography University of California San Diego La Jolla California USA; ^2^ Bermuda Institute of Ocean Sciences Arizona State University St. George's Bermuda

**Keywords:** biogeography, metabarcoding, species distributions, traits, zooplankton

## Abstract

Due to historical under‐sampling of the deep ocean, the distributional ranges of mesopelagic zooplankton are not well documented, leading to uncertainty about the mechanisms that shape midwater zooplankton community composition. Using a combination of DNA metabarcoding (18S‐V4 and mtCOI) and trait‐based analysis, we characterized zooplankton diversity and community composition in the upper 1000 m of the northeast Pacific Ocean. We tested whether the North Pacific Transition Zone is a biogeographic boundary region for mesopelagic zooplankton. We also tested whether zooplankton taxa occupying different vertical habitats and exhibiting different ecological traits differed in the ranges of temperature, Chl‐*a*, and dissolved oxygen conditions inhabited. The depth of the maximum taxonomic richness deepened with increasing latitude in the North Pacific. Community similarity in the mesopelagic zone also increased in comparison with the epipelagic zone, and no evidence was found for a biogeographic boundary between previously delineated mesopelagic biogeochemical provinces. Epipelagic zooplankton exhibited broader temperature and Chl‐*a* ranges than mesopelagic taxa. Within the epipelagic, taxa with broader temperature and Chl‐*a* ranges also had broader distributional ranges. However, mesopelagic taxa were distributed across wider dissolved oxygen ranges, and within the mesopelagic, only oxygen ranges covaried with distributional ranges. Environmental and distributional ranges also varied among traits, both for epipelagic taxa and mesopelagic taxa. The strongest differences in both environmental and distributional ranges were observed for taxa with or without diel vertical migration behavior. Our results suggest that species traits can influence the differential effects of physical dispersal and environmental selection in shaping biogeographic distributions.

## Graphical abstract

The mechanisms shaping midwater zooplankton community composition are uncertain. We used a strong environmental gradient across the North Pacific Transition Zone to test the effects of temperature, Chl‐*a*, and dissolved oxygen concentrations on epipelagic and mesopelagic species distributions. We found broader species distributions and greater community similarity at mesopelagic depths. Variability in environmental ranges suggests that species traits can affect the relative contributions of physical dispersal and environmental selection to biogeographic distributions.

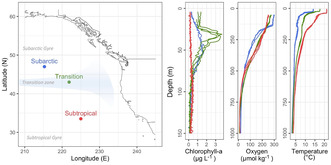

## 1 INTRODUCTION

The mesopelagic zone of the ocean is understudied compared to the epipelagic zone. Despite growing interest in the deep ocean, biodiversity and ecosystem structure in the mesopelagic zone are not well documented. Analyses of environmental climatology have defined 13 mesopelagic biogeochemical provinces with variable vertical extents, most of which occur in multiple ocean basins (Reygondeau et al., 2018). An alternative classification of mesopelagic biogeography used a combination of water masses, environmental climatology, and biotic partitioning to define 33 mesopelagic ecoregions (Sutton et al., 2017). While the biogeographic distributions of pelagic species do not always map cleanly onto biogeochemical provinces, there tend to be distinct pelagic communities in different provinces even when individual species are distributed across multiple provinces (Reygondeau & Dunn, 2018). Despite the disparity in the total number of provinces, there is good agreement in the location of mesopelagic biogeographic and biogeochemical boundaries in the North Pacific (Reygondeau et al., 2018; Sutton et al., 2017). The basin‐scale structure of mesopelagic biogeochemical provinces is broadly similar to that of overlying epipelagic biogeochemical provinces, but the biogeochemical boundaries in the mesopelagic zone may be more permeable to species distributions than observed in the epipelagic zone (Longhurst, 2007; Sutton et al., 2017).

In the North Pacific, differences in epipelagic zooplankton community composition are well documented among biogeochemical provinces, with many zooplankton species restricted to the North Pacific Transition Zone (NPTZ), the Subpolar Gyre, or the Subarctic Gyre (Costello et al., 2017; McGowan & Williams, 1973). Within the epipelagic zone, the NPTZ, which coincides with the eastward‐flowing West Wind Drift, is a distinct biogeochemical province and is home to a characteristic zooplankton community with moderate levels of endemism (Pearcy, 1991). This community is supported by enhanced primary production due to mesoscale mixing within this hydrodynamically complex region (Miyamoto et al., 2022). Within the mesopelagic zone, there are also distinct Subarctic and Subtropical Provinces, but no intervening Transition Province has been recognized (Reygondeau et al., 2018; Sutton et al., 2017). The degree to which the border between Subarctic and Subtropical biogeochemical provinces at midwater depths acts as a biogeographic boundary for mesopelagic zooplankton is unknown, as are the mechanisms by which species distributions might be limited in this region.

Among global ocean biogeochemical provinces, the extent of differentiation between zooplankton communities can vary. Physical dispersal by ocean circulation can increase connectivity among planktonic communities, and genetic breaks in population connectivity often co‐occur with hydrographic barriers to dispersal (Cornils et al., 2017; González et al., 2020). Physical circulation and mixing are slower in the mesopelagic zone than in the epipelagic zone (Kawabe & Fujio, 2010; Reid et al., 1978), which could decrease connectivity and promote geographic divergence among zooplankton communities. However, biogeographic distributions are also shaped by habitat suitability (Goetze et al., 2017). While physical transport can introduce expatriates to new locations, in suboptimal habitats, these individuals may not establish autochthonous populations. Compared to the epipelagic zone, the mesopelagic zone has significantly lower variability in temperature and food availability, particularly at basin scales and after averaging short‐term mesoscale variability (Robinson et al., 2010). Dissolved oxygen and pH may be the exception to this rule, with significant oxyclines occurring within the mesopelagic zone in many pelagic environments. However, the overall greater environmental homogeneity in the mesopelagic zone leads to a contrasting prediction: that there could be broader species distributions and greater community similarity at mesopelagic than epipelagic depths.

The biogeographic distributions of mesopelagic zooplankton will thus reflect the combined influences of greater environmental homogeneity and decreased physical mixing relative to the surface ocean. The relative importance of each may vary with species‐specific physiological tolerances and behaviors. Differences in ecological strategies and environmental ranges tolerated may lead to variability in the effects of dispersal and environmental heterogeneity. In the trait‐based approach, species‐specific traits that influence a species' ability to feed, survive predation, or reproduce can be used to identify potential trade‐offs in ecological strategies among taxa (Litchman et al., 2013). Individual characteristics such as body size can be compared across taxa to identify emergent general principles (Ohman & Romagnan, 2016), or combinations of traits can be used to identify functional groups that share ecological strategies and have similar ecosystem functions (Venello et al., 2021). Within zooplankton, trait‐based analyses have primarily been applied to copepods, leading to the characterization of functional groups that have similar ecological responses to environmental changes, and identification of latitudinal gradients in feeding behavior, diet, and body size, among other traits (Benedetti et al., 2021; Brun et al., 2016). Trait‐based analyses can be strengthened by comparing traits across multiple major taxonomic groups, expanding the range of trait states considered.

DNA metabarcoding uses targeted amplicon sequencing of bulk zooplankton samples to characterize zooplankton community composition (Bucklin et al., 2016). Primers are designed to amplify sequences from multiple species at specific marker regions, and so‐called “universal primers” can resolve a broad array of taxonomic groups. The inclusion of multiple marker regions can further increase taxonomic breadth and resolution (Questel et al., 2021). While primer biases can lead to skewed representations of total community composition, comparison of read proportions across samples can reasonably approximate changes in species proportions (Matthews et al., 2021). Detection sensitivity can be quite high, making metabarcoding particularly useful for the inclusion of rarer species. When used in combination with reference sequence databases and trait databases, metabarcoding sequences can be identified taxonomically and then linked to species traits.

Here, we analyze zooplankton community composition between the surface and 1000 m in the Subarctic Gyre, the NPTZ, and the Subtropical Gyre in order to compare epipelagic and mesopelagic biogeographic distributions across this region. We test (1) whether the NPTZ is a biogeographic boundary region for mesopelagic zooplankton communities; (2) whether epipelagic and mesopelagic taxa have different biogeographic and environmental ranges in the Northeastern Pacific Ocean; and (3) whether biogeographic and environmental ranges vary among zooplankton with different traits.

## 2 MATERIALS AND METHODS

### 2.1 Sampling

**FIGURE 1:**
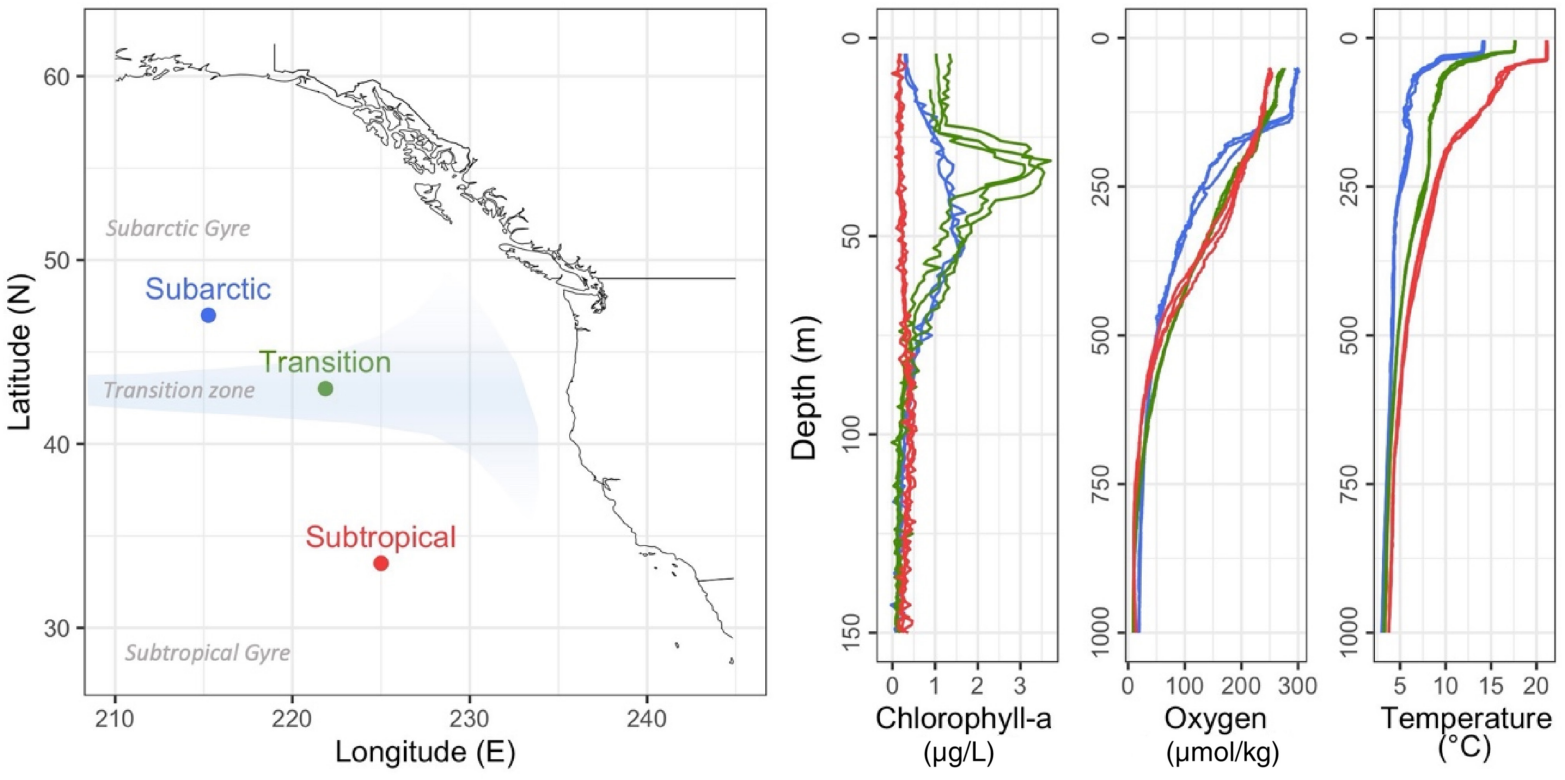
Locations of MOCNESS tows in the Northeast Pacific and CTD profiles of chlorophyll‐a fluorescence, temperature, and dissolved oxygen at each of the sampling locations (red: Subarctic Province; blue: Transition Province; green: Subtropical Province). Major oceanographic regions are labeled in gray. Chl‐a fluorescence profiles are shown from 0 to 150 m, and oxygen and temperature profiles from 0 to 1000 m. Ocean circulation features from Reid et al. (1978).

Samples were collected in the Northeast Pacific Ocean between August 8 and September 18, 2012, onboard the R/V *New Horizon* (Figure 1). At each sampling location, one daytime and nighttime MOCNESS tow (Wiebe et al., 1985) was conducted across the epipelagic (0–200 m) and mesopelagic (200–1000 m) zones of the ocean, with 8 discrete depth strata: 1000–800 m, 800–600 m, 600–400 m, 400–200 m, 200–100 m, 100–50 m, 50–25 m, and 25–0 m. The net mesh size was 150 μm and the net area was 1 m^2^. Upon recovery, samples were held on ice, and large fish were fixed and stored separately. On some samples, a few (<10 per sample; in general, one per sample) pteropods and euphausiids were removed before splitting and preservation (see https://www.bco‐dmo.org/dataset/3546). The sample was then quantitatively split with a Motoda box‐splitter. One half of the sample was preserved in 95% ethanol, one quarter in 5% buffered formalin, and one quarter in 70% non‐denatured ethanol.

At each sampling location, hydrographic profiles were collected as a part of standard casts with a CTD rosette. The rosette included sensors for temperature, conductivity, and depth (Digiquartz), as well as for dissolved oxygen (SBE43, SeaBird Electronics), a transmissometer (Wet Labs C*, 660 nm wavelength), and a chlorophyll‐*a* fluorometer (Wet Labs ECO‐AFL). Vertical profiles were collected on continuous downcasts (to 1000 m), and all data were averaged into 1‐m vertical bins for subsequent calculations of environmental ranges and environmental means in discrete MOCNESS sampling depths.

### 2.2 Extraction, amplification, and sequencing

DNA extraction followed previously published protocols for ethanol‐preserved bulk zooplankton tissue (Blanco‐Bercial, 2020). In short, after removal of ethanol and milliQ washing, the zooplankton pellet was weighted and transferred to 15–25 mL of SDS buffer (Tris–HCl 10 mM, EDTA 100 mM pH 8.0, NaCl 200 mM, SDS 1%). Samples were vortexed to break up the pellet and then ground using a Fisher Scientific Homogenizer FSH125. After the grinding protocol, proteinase K (Sigma‐Aldrich, St. Louis, MO) was added to the buffer (0.2 mg/mL final concentration), as well as 5 mL of sterilized stainless‐steel Shot, 3/32″ Ellipse (RioGrande, CA). The tubes were incubated at 60°C for 4 h, vortexing every 30 min, then centrifuged to pellet the tissue. Three (pseudo)replicates of 400 μL of the supernatant were transferred to 1.5 mL Eppendorf tubes. After this step, DNA was extracted following the E.Z.N.A.® Mollusk DNA Kit (Omega Bio‐tek, Norcross, GA), from the addition of chloroform:isoamyl alcohol 24:1 step onward.

Concentrations of extracted DNA were normalized in deionized water to concentrations of 4 ng/μL. Each sample was amplified in triplicate using the 18S‐V4 (primers Uni18S/Uni18SR) (Zhan et al., 2013) and mtCOI (primers mlCOIintF/jgHCO2198) (Leray et al., 2013) marker regions. This combination of molecular markers allows for increased taxonomic breadth (i.e., detection of more taxa) while still allowing for discrimination of closely related species (Questel et al., 2021). Libraries were prepared using a combined two‐step PCR and library preparation protocol, as described previously (Matthews et al., 2021). Duplicate dual indexes were used for each sample, with independent indexes for PCR replicates. Final PCR reactions were cleaned with AMPure XP magnetic beads (Beckman Coulter), and DNA concentrations were quantified with Picogreen assays. Samples were pooled in equimolar concentrations within each marker region, with 20% higher concentrations for the 18S marker to compensate for preferential sequencing of the shorter COI sequences. The pooled library was sequenced on an Illumina MiSeq with 300 bp paired‐end V3 chemistry (IGM Genomics Center, University of California San Diego, La Jolla, CA).

### 2.3 Bioinformatic processing

Bioinformatic processing followed previously described pipelines (Matthews et al., 2021). In brief, samples were demultiplexed based on unique combinations of forward and reverse indexes, with only sequences with perfect matches to all four indexes retained. Demultiplexed sequences were visually assessed for quality, then trimmed and denoised into ASVs with *dada2* in QIIME2 (Bolyen et al., 2019), using pseudo‐pooling to minimize false positives (Callahan et al., 2017). COI data were subsequently clustered into OTUs at 97% similarity using *vsearch* in QIIME2. Sequences recovered from negative controls were used to remove potential contaminants through the R package *decontam* (Davis et al., 2018). Technical replicates were visually assessed based on total richness, taxonomic composition, and nMDS clustering. As all replicates with successful PCRs were similar, replicates for each unique biological sample were merged by summing the total reads from each technical replicate. Taxonomic assignments for 18S ASVs and COI OTUs were made with the *sklearn* classifier implemented in QIIME2. The classifier was trained on a custom zooplankton database created from the global MetaZooGene COI and 18S databases v2022‐10‐26 (Bucklin et al., 2021), supplemented with 242 COI sequences and 283 18S sequences collected from 53 zooplankton species identified from the southern California Current System (Table S1). Prior to classifier training, all sequences were trimmed with appropriate primers using virtual PCR implemented with the R package *insect*, with primer regions retained. Taxonomic assignments were retained if they had at least 80% confidence levels. All species‐level classifications were manually compared against previously described biogeographic distributions for the species (Horton et al., 2022; Razouls et al., 2005; The Deep‐Sea Guide (DSG), 2023). If sequences were identified as species that do not occur in the North Pacific, the taxonomic assignment was only retained at the genus level. Sequences unidentified by phylum (including any prokaryotic sequences) were removed from the analysis, as were all protistan, mammal, and fish sequences.

### 2.4 Trait assignment

Traits were mapped to taxonomic assignments as previously described (Matthews & Ohman, 2023), with traits defined both by taxon‐specific trait databases (Benedetti et al., 2016; Brun et al., 2016) and from the primary literature. The following traits were assigned where possible: body size (maximum reported for the species), diet (carnivore, omnivore, or herbivore; detritivores were included as omnivores), feeding behavior (ambush, cruise, parasitoid, or suspension), spawning strategy (broadcast spawning or brooding), presence of asexual reproduction (yes, no), body composition (gelatinous, intermediate, or non‐gelatinous), and Diel Vertical Migration behavior (DVM) (yes, no). The presence of DVM was determined from day to night differences in sequence reads, according to the following criteria: if the taxon was observed in at least two nets in each tow, the total relative abundances in the day and night tows were no more than twofold different, the taxon comprised at least 0.01% of the total read abundance in the study, and the weighted mean depth of occurrence shifted by at least two sampling strata. For species with spatial or temporal plasticity (e.g., species with a preference for herbivory but the capacity for omnivory), we used the most inclusive trait. For each unique taxon that we observed, we assigned all traits that were possible for the specificity of the taxonomic assignment. For genera and families with consistent traits among all species, traits were assigned even if the amplicon was unidentified at the species level; for taxonomic groups with variable traits, that trait was not assigned, and the amplicon was not included in trait‐based comparisons.

### 2.5 Statistical analyses

All statistical analyses were conducted in R 4.2.1 (2022‐06‐23), primarily using base R  as well as the packages *vegan*, *phyloseq*, *ggpubr*, and *tidyverse*. Taxonomic richness was calculated from presence. All comparisons of richness were conducted in parallel with rarefied and non‐rarefied datasets. For all subsequent statistical analyses after comparisons of richness, ASV and OTU tables were converted to relative abundance but were not rarefied (McMurdie & Holmes, 2014; see also Schloss, 2020). Mean depth of occurrence (MDO) was calculated for each taxon by averaging net midpoints for all nets in which the taxon was observed, weighted by relative read abundance within the net. MDO was calculated independently for each MOCNESS tow as well as overall across all sampling locations. Taxon body size and MDO were tested for normality using Shapiro–Wilk tests and found to be non‐normally distributed even after logarithmic or square‐root transformations, so correlations of untransformed data were tested using ties‐corrected non‐parametric Spearman rank correlation. To visualize community similarity among samples, non‐metric multidimensional scaling was used as amplicon data are non‐normally distributed and relative abundances are not independent of each other (Ramette, 2007). Beta‐dispersion was calculated from Bray–Curtis distance and Jaccard distance, and within‐group dispersions for epipelagic (0–200 m) and mesopelagic (200–1000 m) samples were compared with a one‐way analysis of variance (Anderson et al., 2006).

All CTD values from each net depth range within a province were averaged to describe the mean conditions for each sample. The environmental range inhabited by each taxon was calculated from the difference between the maximum and minimum of the mean environmental property for the nets in which the taxon occurred. To allow for uneven sample sizes among groups and non‐normal distributions of environmental ranges, non‐parametric Kruskall–Wallis rank sum tests were used to test for differences in environmental and biogeographic ranges. For comparisons with more than two groups, significant differences between groups were identified with a two‐sided post‐hoc Dunn's test of multiple comparisons with a Bonferroni adjustment for family‐wise error rate. For ease of interpreting visualizations, ranges were plotted with 20% scatter around categorical values and 0% scatter around numerical values. All data analysis scripts, including bioinformatic processing, statistical analyses, and plotting, are available at https://github.com/samatthews/NorthPacificMidwater/.

## 3 RESULTS

In total, we recovered 4.05 million COI sequences and 2.44 million 18S sequences, of which 2.56 million (COI) and 2.34 million (18S) passed quality control and were identified as zooplankton (Table S2). After bioinformatic processing and removal of contaminants and non‐zooplankton sequences, 718 OTUs were detected at the COI marker (1.8 × 10^6^ reads, 97% similarity clustering) and 491 ASVs at the 18S marker (2.3 × 10^6^ reads). Average sequencing depth per biological sample after all quality control and filtering was 5.1 × 10^4^ reads (7.4 × 10^3^–1.3 × 10^5^) at the COI marker and 4.6 × 10^4^ reads (9.5 × 10^3^–8.9 × 10^4^) at the 18S marker. We found no relationship between sequencing depth and richness at the COI marker, and a negative relationship at the 18S marker (Figure S1).

### 3.1 Zooplankton community composition

**FIGURE 2:**
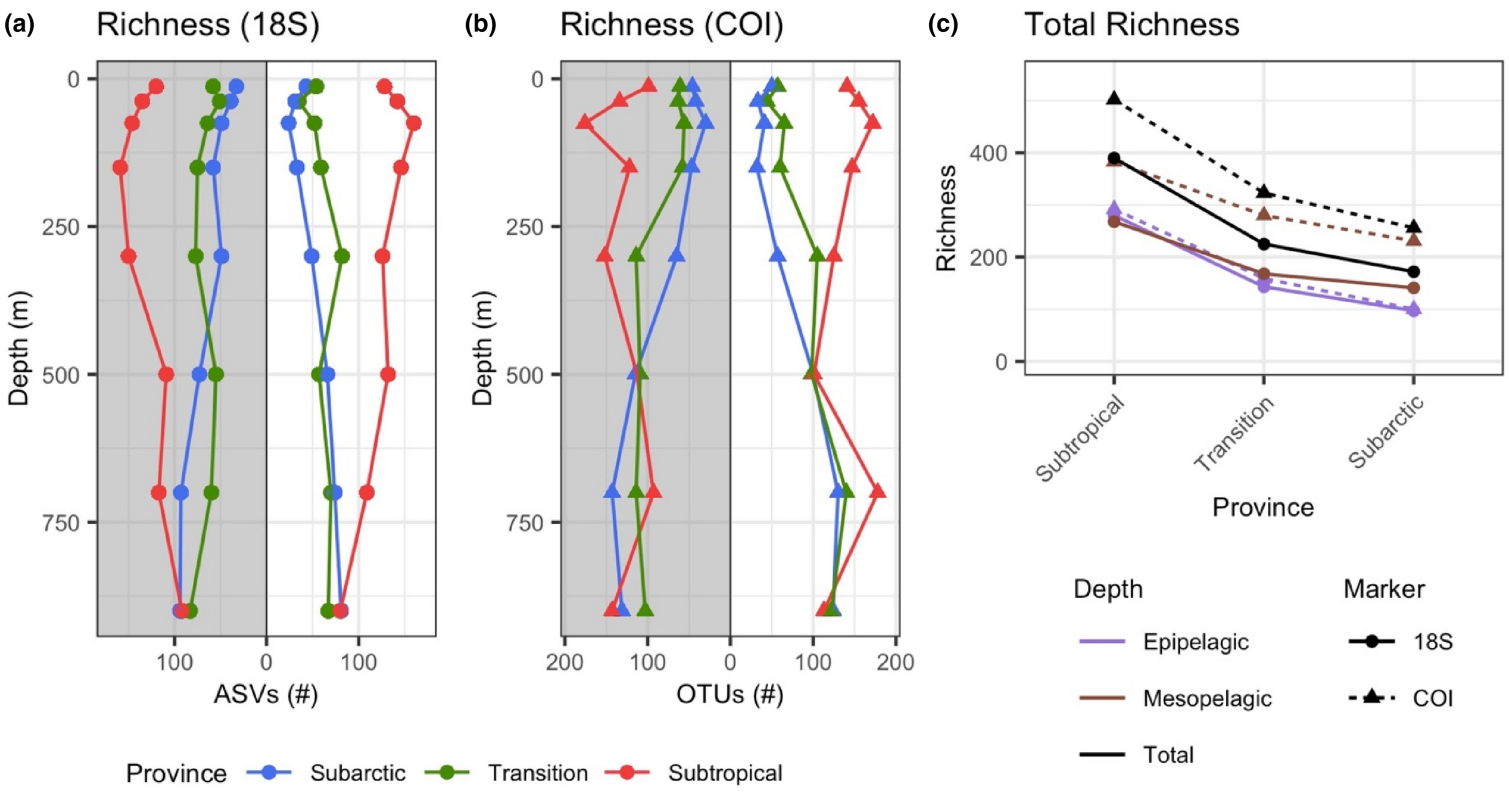
Taxonomic richness across the three biogeographic provinces for (a) 18S ASVs across depth, (b) COI OTUs across depth, and (c) total richness within the epipelagic zone, mesopelagic zone, and across the full 0–1000 m sampled. For panels (a, b), points are plotted at the midpoint of each discrete zooplankton net range, with nighttime samples on the left (gray background) and daytime samples on the right (white background). For panel (c), colors indicate depth zones, and line types denote marker. Richness is the total number of ASVs/OTUs for 18S or COI, respectively.

Despite higher overall richness in the COI‐resolved community, vertical and horizontal patterns in richness were similar at the two markers (Figure 2a,b). In the Subtropical Province, richness peaked in the 50–100 m net and decreased with depth, apart from a secondary peak in the 600–800 m net in the daytime COI samples (Figure 2b). In contrast, vertical richness in the Transition and Subarctic Provinces was lowest between the surface and 50–100 m, then increased with depth, both day and night. The richness maxima in both the Transition and Subarctic Provinces were observed in the 600–800 m and 800–1000 m nets. In total, zooplankton richness within the upper 1000 m was highest in the Subtropical Province, moderate within the Transition Province, and lowest in the Subarctic Province (Figure 2c). Richness patterns were similar for rarefied data, confirming that these patterns are not artifacts of sequencing depth (Figure S2).

**FIGURE 3:**
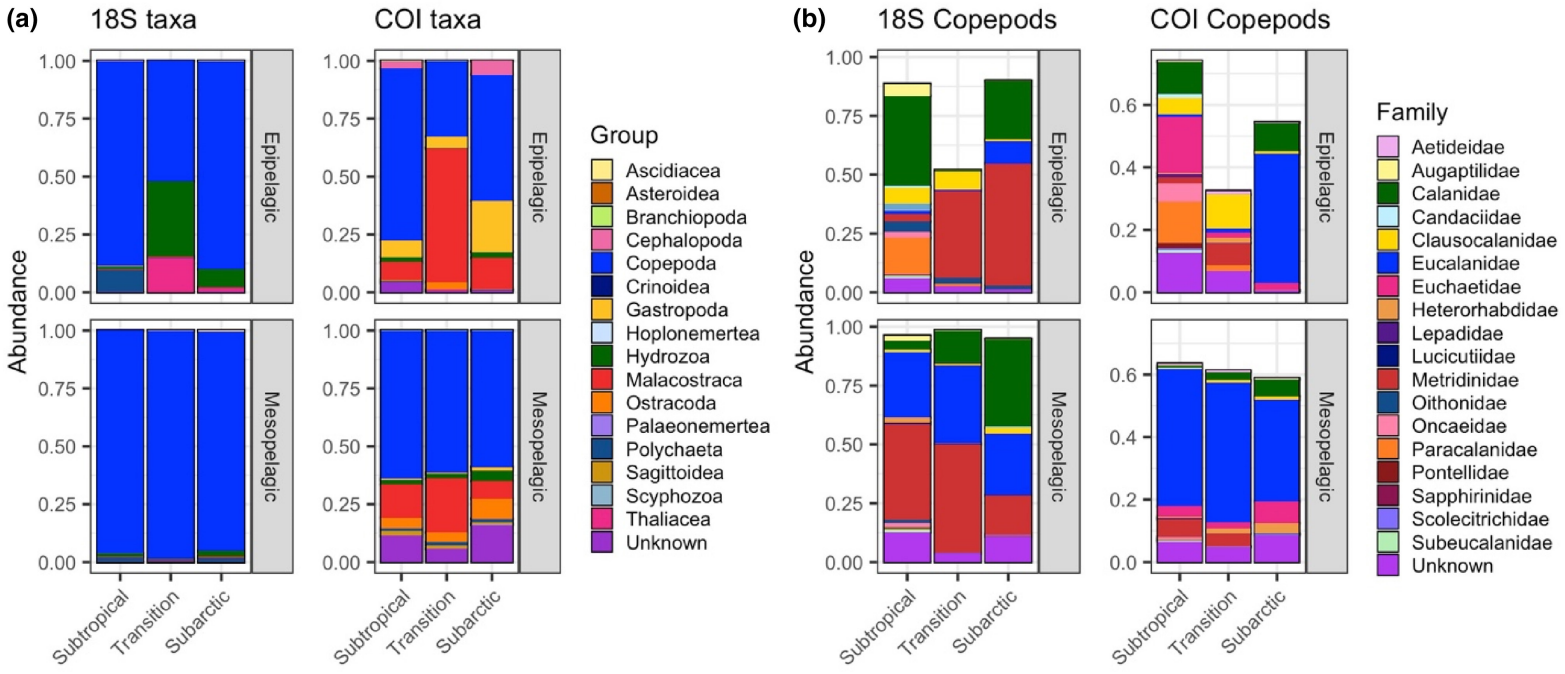
Relative read abundance of (a) taxonomic classes and (b) families within the class Copepoda. Relative read abundance is defined as a proportion of all COI reads (left column of each panel) and all 18S reads (right column of each panel) across day and night samples. Colors represent taxonomic groups. Epipelagic samples (0–200 m) are plotted in the upper subpanel of each set, and mesopelagic samples (200–1000 m) in the lower subpanels.

Across all depths and all provinces, the zooplankton community was dominated by the class Copepoda (Figure 3a). The lowest relative abundance of copepods was observed in the epipelagic zone of the Transition Province, both at 18S and COI. The epipelagic Transition Province was characterized by relatively higher proportions of hydrozoans (18S), thaliaceans (18S), and malacostracans (COI). Within the class Copepoda, there were notable shifts among provinces in the relative abundance of taxonomic families within the epipelagic community (Figure 3b, upper panels). The highest diversity of copepod families (24 families between both markers) was observed within the Subtropical epipelagic zone, which was comprised primarily of Calanidae and Paracalanidae, followed by Euchaetidae and Clausocalanidae. Clausocalanidae were also relatively abundant in the epipelagic Transition Province, although in lower proportions than Metridinidae. In the Subarctic Province, Metridinidae had the largest proportion of epipelagic copepod reads, followed by Eucalanidae and Calanidae. In the mesopelagic zone, copepod community composition was less variable among provinces (Figure 3b, lower panels). Eucalanidae were the most abundant group in all three provinces, followed by Metridinidae and Calanidae. The relative contributions of Metridinidae and Calanidae reads varied across provinces, with more Metridinidae in the Subtropical Province mesopelagic zone, a mixture of Metridinidae and Calanidae in the Transition Province mesopelagic zone, and greater proportions of Calanidae in the mesopelagic zone of the Subarctic Province.

**FIGURE 4:**
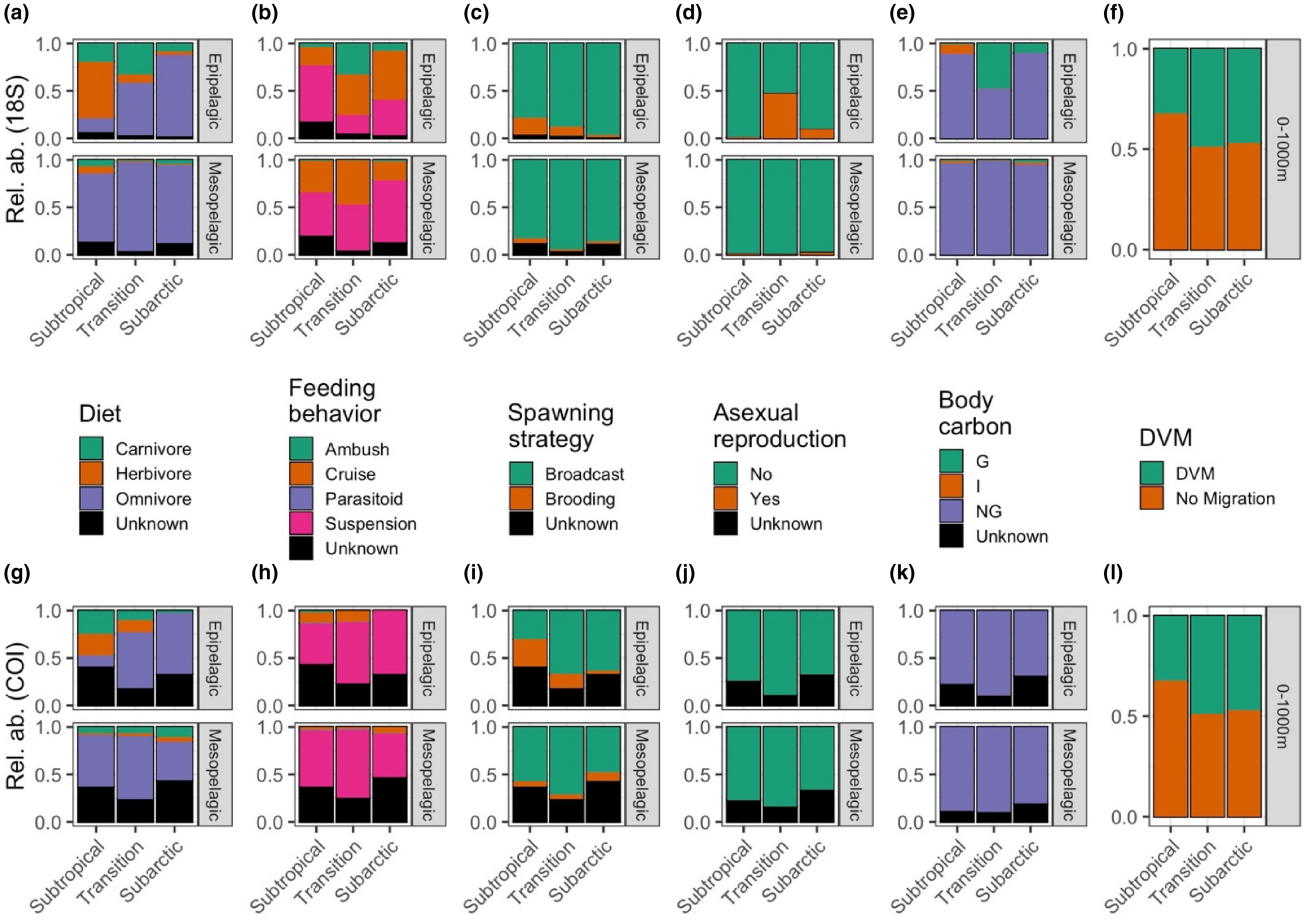
Relative read abundance assigned to each trait state at the 18S marker (upper row) and COI marker (lower row), within the epipelagic zone (upper subpanels within each row) and mesopelagic zone (lower subpanels within each row) for (a, g) dietary preference; (b, h) feeding behavior; (c, i) spawning strategy; (d, j) presence or absence of asexual reproduction; (e, k) body carbon composition (G: gelatinous; I: intermediate; NG: non‐gelatinous); and (f, l) presence or absence of detectable DVM behavior. DVM behavior is not divided into vertical strata, as animals migrate between strata.

For the traits of diet, feeding behavior, spawning strategy, presence of asexual reproduction, and body carbon composition, we observed more variability in the relative abundance of trait states among biogeographic provinces within the epipelagic zone than within the mesopelagic zone (Figure 4). Within the epipelagic zone, diets shifted from a carnivore‐ and herbivore‐dominated community in the Subtropical Province to an almost entirely omnivorous community in the Subarctic Province, while the mesopelagic community consisted primarily of omnivores in all three provinces (Figure 4a,g, upper panels). The most common feeding behavior across all three provinces and both depth zones was suspension feeding, followed by moderate levels of cruise feeding, but ambush feeding was relatively more common in the epipelagic than in the mesopelagic and peaked in relative abundance in the Transition Province (Figure 4b,h). Brooding behavior and asexual reproduction were both more common in the epipelagic zone than in the mesopelagic zone. Brooding was primarily observed in the epipelagic zone of the Subtropical Province and, to a lesser extent, in the epipelagic zone of the Transition Province (Figure 4c,i, upper panels). Asexual reproductive strategies were primarily observed in the epipelagic zone of the Transition Province and, to a lesser extent, in the epipelagic zone of the Subarctic Province, but only in the 18S data (Figure 4d,j). Most reads were classified as belonging to non‐gelatinous taxa (Figure 4e,k), but at 18S, gelatinous reads comprised approximately one third of all reads in the epipelagic zone of the Transition Province and a small number of reads in the Subarctic Province epipelagic zone (Figure 4e). Intermediate body compositions were primarily observed in the Subtropical Province within the epipelagic zone. Approximately 50% of all reads belonged to taxa with detectable DVM behavior, and in the Subtropical Province there was a slight decrease in the relative abundance of reads that corresponded to taxa with DVM behavior (Figure 4f,l).

**FIGURE 5:**
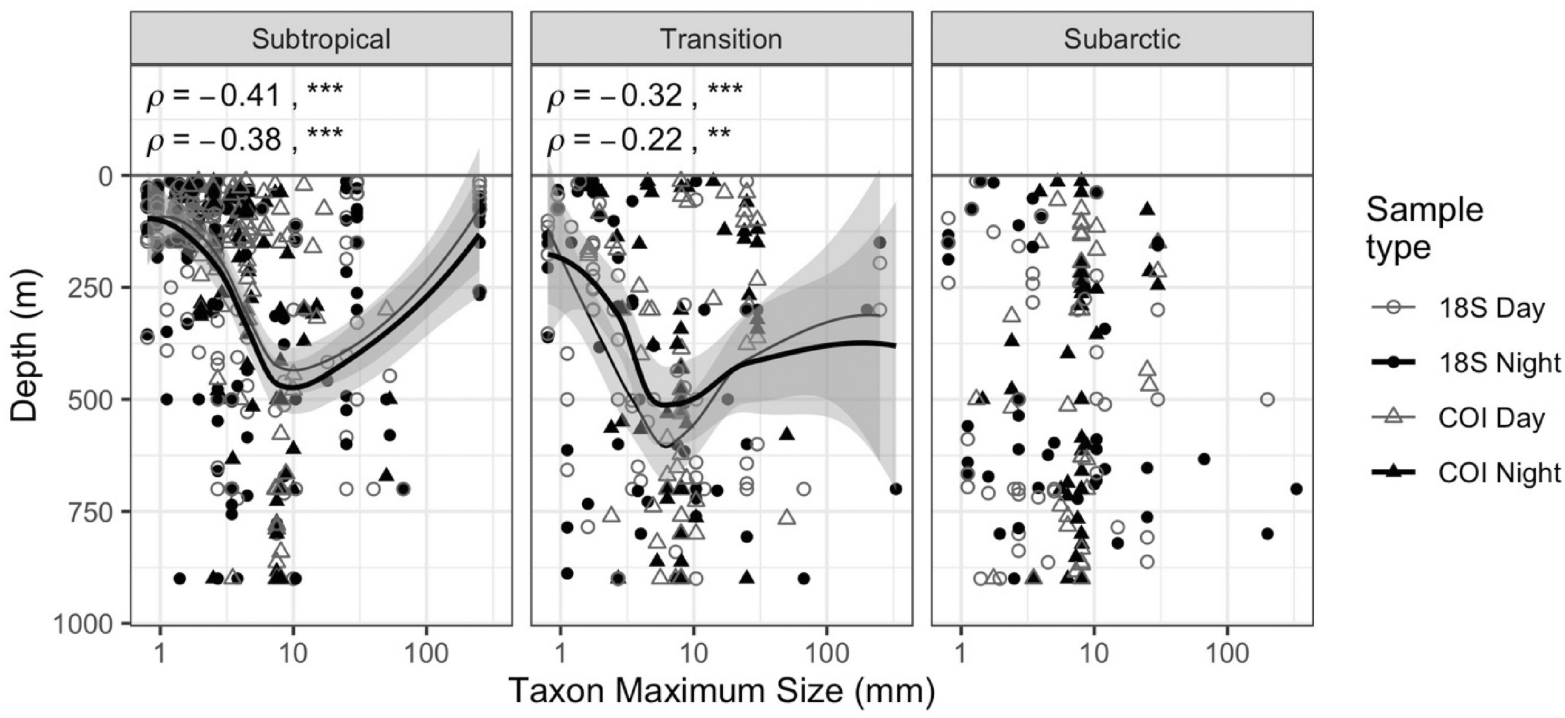
Relationship between the maximum reported body size and observed mean depth of occurrence in each of the three provinces Loess best‐fit lines are shown for the significant relationships observed for mean depth of occurrence in the daytime (open symbols) and nighttime (closed symbols) in the Subtropical and Transition provinces. Shading the surrounding lines represents the 95% CI. The daytime mean depth of occurrence is plotted in gray, and the nighttime mean depth of occurrence is black. Circles denote taxa detected with 18S and triangles denote taxa detected with COI (Spearman's rank: ns = *p* > .05, *p < .05, **p < .01, ***p < .001).

Taxon body size was significantly correlated with mean depth of occurrence for both daytime and nighttime vertical distributions in the Subtropical and Transition Provinces (ties‐corrected Spearman rank correlation; Subtropical Province day: *ρ =* 0.38, *p* < .0001; Subtropical Province night: *ρ =* 0.41, *p* < .0001; Transition Province day: *ρ =* 0.22, *p* < .0001; Transition Province night: *ρ =* 0.32, *p* < .0001; Figure 5). A *loess* non‐parametric locally fitted regression showed that the positive correlations between size and depth were restricted to taxa smaller than 10 mm. In the Subarctic Province, we found no significant relationship between the maximum reported taxon size and the observed mean depth of occurrence. Among provinces, we found no significant differences in epipelagic or mesopelagic taxon body sizes (Kruskal–Wallis rank sum test *p* > .05, both depth zones). We also tested whether size was correlated with mean depth of occurrence within major zooplankton groups and found larger taxa at deeper depths for copepods (Class Copepoda) and euphausiids (Class Malacostraca) (Figure S3).

### 3.2 Taxa distributions across provinces

**FIGURE 6:**
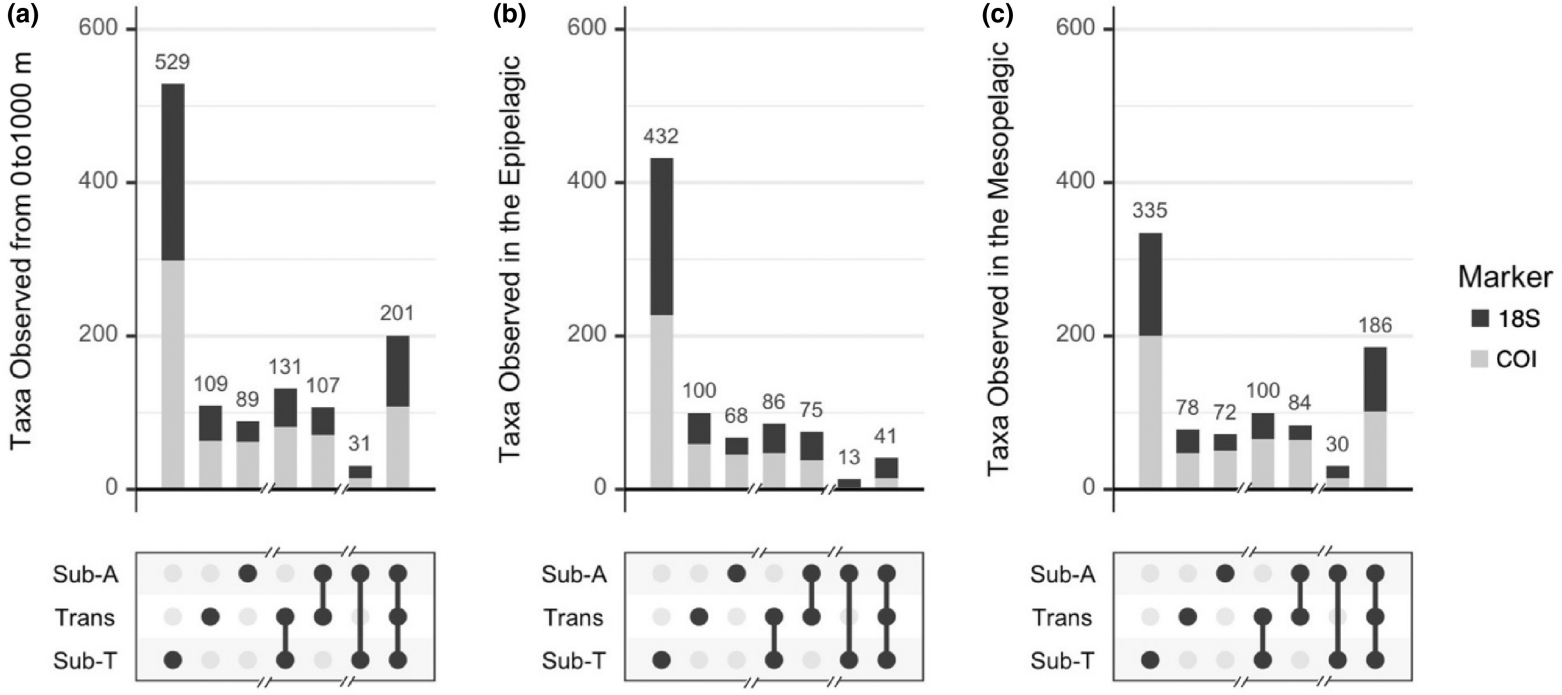
Number of taxa (OTUs and ASVs) observed within only one province or shared between multiple provinces for (a) the full water column (0–1000 m), (b) the epipelagic zone (0–200 m), and (c) the mesopelagic zone (200–1000 m). Black shading represents taxa detected with 18S, and gray shading represents taxa detected with COI. Bar heights in the upper panel represent the total number of taxa observed within each province or combination of provinces. Filled circles and connecting lines in the lower panel represent the province or provinces in which the taxa were observed. Sub‐a, Subarctic Province; Trans, Transition Province; Sub‐T, Subtropical Province.

We examined whether taxa were observed in a single province, distributed across two provinces, or extended across all three biogeographic provinces (Figure 6). Across the full 0–1000 m sampled, the largest group of taxa in our study was observed only in the Subtropical Province (529), followed by taxa observed across all three provinces (201) (Figure 6a). More taxa were shared between the Subtropical and Transition Provinces (131) than between the Transition and Subarctic Provinces (107), and the smallest number of taxa were those observed in the Subtropical Province and Subarctic Province but not the Transition Province (31). When we divided the water column into vertical strata, we found increased rates of endemism in the epipelagic zone (35%, 33%, and 76% of taxa in the Subarctic, Transition, and Subtropical Provinces, respectively), compared with the mesopelagic zone (19%, 17%, and 51%). Across the full water column, 17% of all taxa were cosmopolitan, appearing in all three stations. The percentage of cosmopolitan taxa decreased to 5% within the epipelagic zone and increased to 21% within the mesopelagic zone.

**FIGURE 7:**
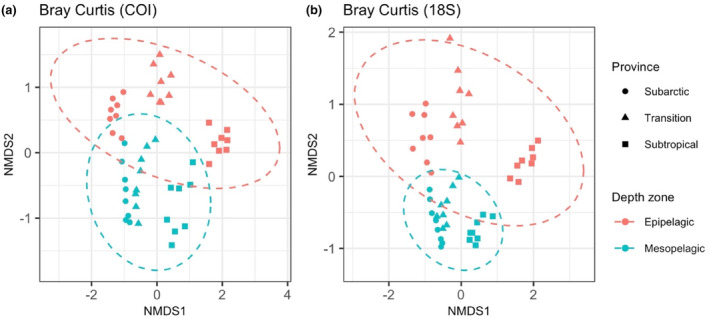
Non‐metric multidimensional scaling of the Bray–Curtis distance between samples, calculated from (a) 18S and (b) COI amplicons. Symbolic shapes indicate different biogeographic provinces, and colors denote vertical depth zones. The dashed ellipses denote the 95% confidence level of the multivariate *t*‐distribution.

When samples were clustered using non‐metric multidimensional scaling of Bray–Curtis distance between samples, mesopelagic samples clustered more closely than epipelagic samples (Figure 7). To test whether community similarity across the three provinces was higher in the mesopelagic than in the epipelagic, we calculated the beta‐dispersion within samples from each depth zone. Mesopelagic samples had lower within‐group beta‐dispersion, confirming that mesopelagic communities exhibit fewer differences across provinces than epipelagic communities (18S: *F*(1, 46) = [12.039], *p* < .01, Figure 7a; COI: *F*(1, 46) = [5.0466], *p* < .05, Figure 7b). Parallel analyses using Jaccard distance calculated from presence/absence similarly found higher similarity among mesopelagic samples than among epipelagic samples (18S: *F*(1, 46) = [9.123], *p* < .01; COI: F(1, 46) = [4.8572], *p* < .05; not shown).

**FIGURE 8:**
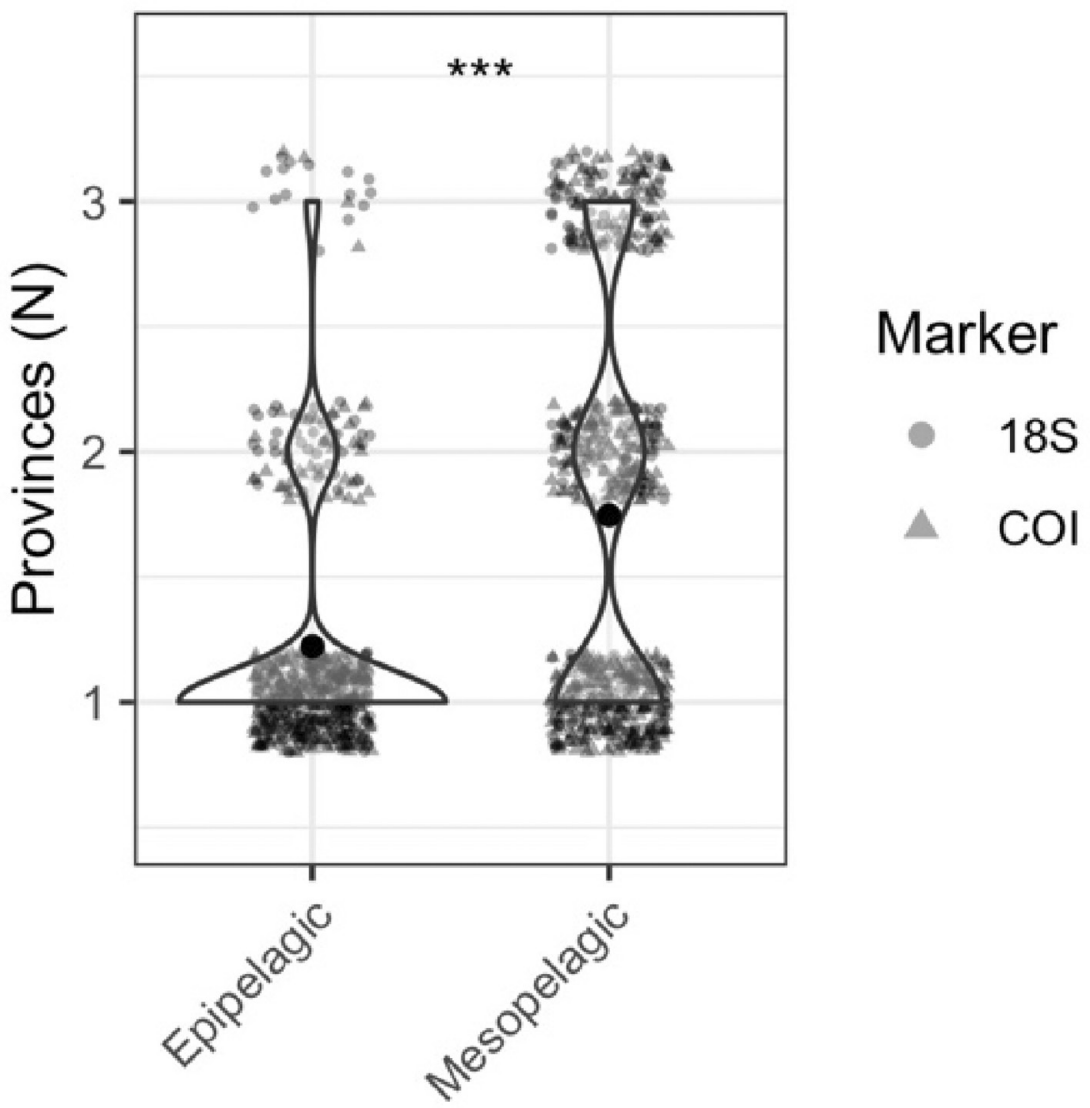
Epipelagic and mesopelagic OTUs and ASVs, and the number of provinces in which each taxon was observed. Violin plots show a density distribution with data points jittered within each categorical value. Data points are semi‐opaque; variability in color intensity reflects overlapping points. Circles denote taxa detected with 18S, and triangles denote taxa detected with COI (Kruskal–Wallis: ns = *p* > .05, *p < .05, **p < .01, ***p < .001).

In addition to dividing the zooplankton community into discrete epipelagic and mesopelagic depth zones, we classified each taxon as primarily epipelagic or primarily mesopelagic based on mean read depth across all provinces. Taxa classified as mesopelagic (MDO > 200 m) were significantly more likely to be observed in multiple provinces than taxa classified as epipelagic (MDO < 200 m) (*χ* ^2^ = 152.01, *p* < .001) (Figure 8).

### 3.3 Environmental range and ecological strategies

**FIGURE 9:**
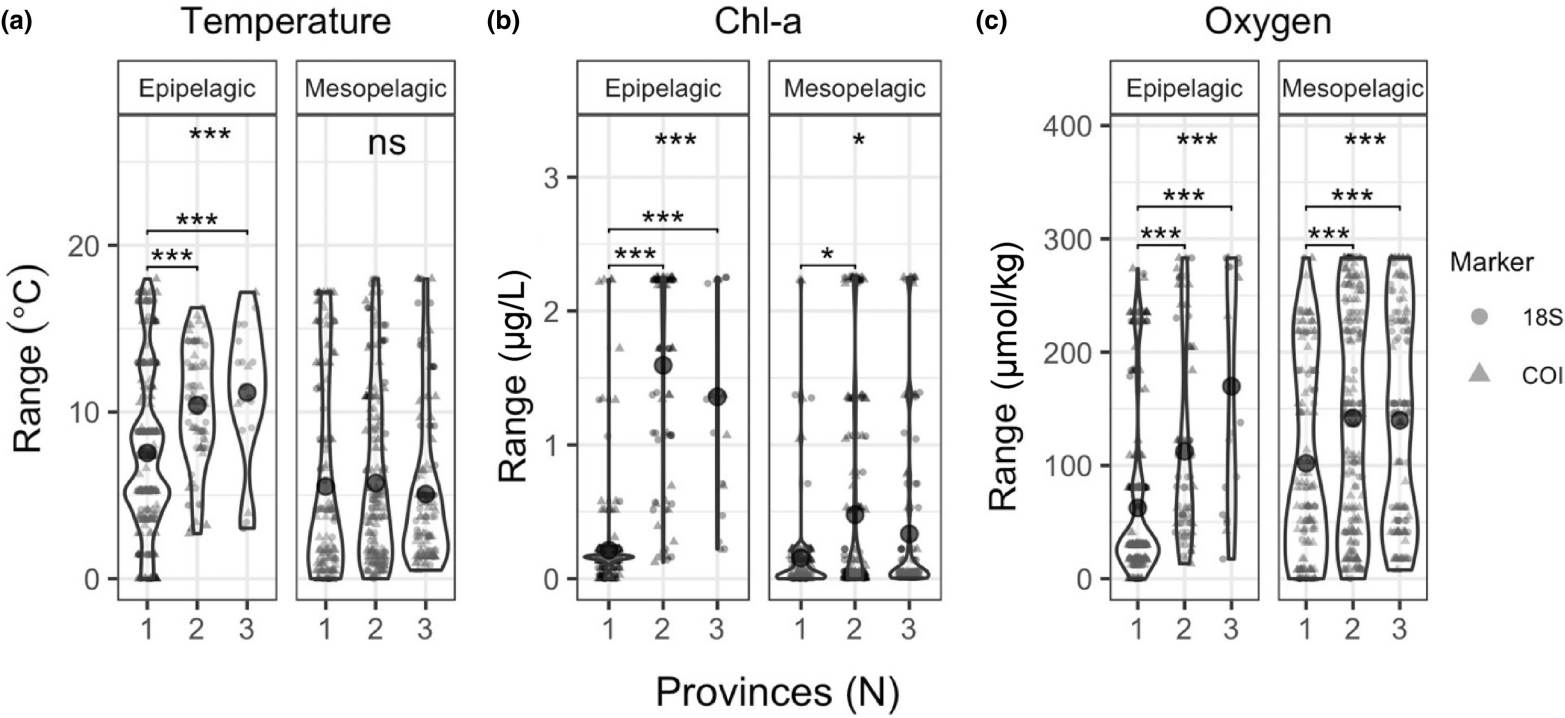
Environmental ranges across which epipelagic and mesopelagic taxa were distributed for taxa observed in one, two, or three biogeographic provinces. (a) temperature ranges; (b) Chl‐a ranges; (c) oxygen ranges. Violin plots show density distribution with data points jittered within each categorical value. Data points are semi‐opaque; variability in color intensity reflects overlapping points. Circles denote taxa detected with 18S, and triangles denote taxa detected with COI. Global significance within each subpanel reflects the results of the Kruskal–Wallis test among taxa observed in 1, 2, or 3 provinces; pairwise comparisons are Bonferoni‐corrected post‐hoc Dunn's test results (ns = *p* > .05, *p < .05, **p < .01, ***p < .001).

We used differences in the mean environment of each sample to test whether there were differences between epipelagic and mesopelagic taxa in the ranges of environmental properties inhabited by each taxon. Kruskal–Wallis tests revealed significantly different ranges for epipelagic and mesopelagic taxa, with larger temperature ranges for epipelagic taxa (*χ* ^2^ = 21.992, 1 df, *p* < .001), larger Chl‐*a* ranges for epipelagic taxa (*χ* ^2^ = 39.292, 1 df, *p* < .001), and larger oxygen ranges for mesopelagic taxa (*χ* ^2^ = 43.982, 1 df, *p* < .001) (Figure S4). To investigate whether these wider environmental ranges are associated with greater biogeographic ranges, we tested whether taxa observed in one, two, or three provinces were distributed across wider ranges of temperature, Chl‐*a*, or dissolved oxygen (Figure 9). We found significant differences in temperature ranges of epipelagic taxa observed in one, two, or three provinces (χ^2^ = 37.715, 2 df, *p* < .001) but no differences among mesopelagic taxa (Figure 9a). The Chl‐*a* ranges of epipelagic taxa observed in only one province were significantly narrower than the Chl‐*a* ranges of taxa observed in two or three provinces (χ^2^ = 174.23, 2 df, *p* < .001), and mesopelagic taxa observed only in a single province had narrower Chl‐*a* ranges than taxa observed in two provinces, but not significantly different ranges than taxa observed in three provinces (χ^2^ = 7.2583, 2 df, *p* < .05) (Figure 9b). Both epipelagic and mesopelagic taxa found in two or three provinces were found across significantly wider oxygen ranges than taxa found only in a single province (epipelagic: χ^2^ = 67.868, 2 df, *p* < .001; mesopelagic: χ^2^ = 17.978, 2 df, *p* < .001) (Figure 9c).

**FIGURE 10:**
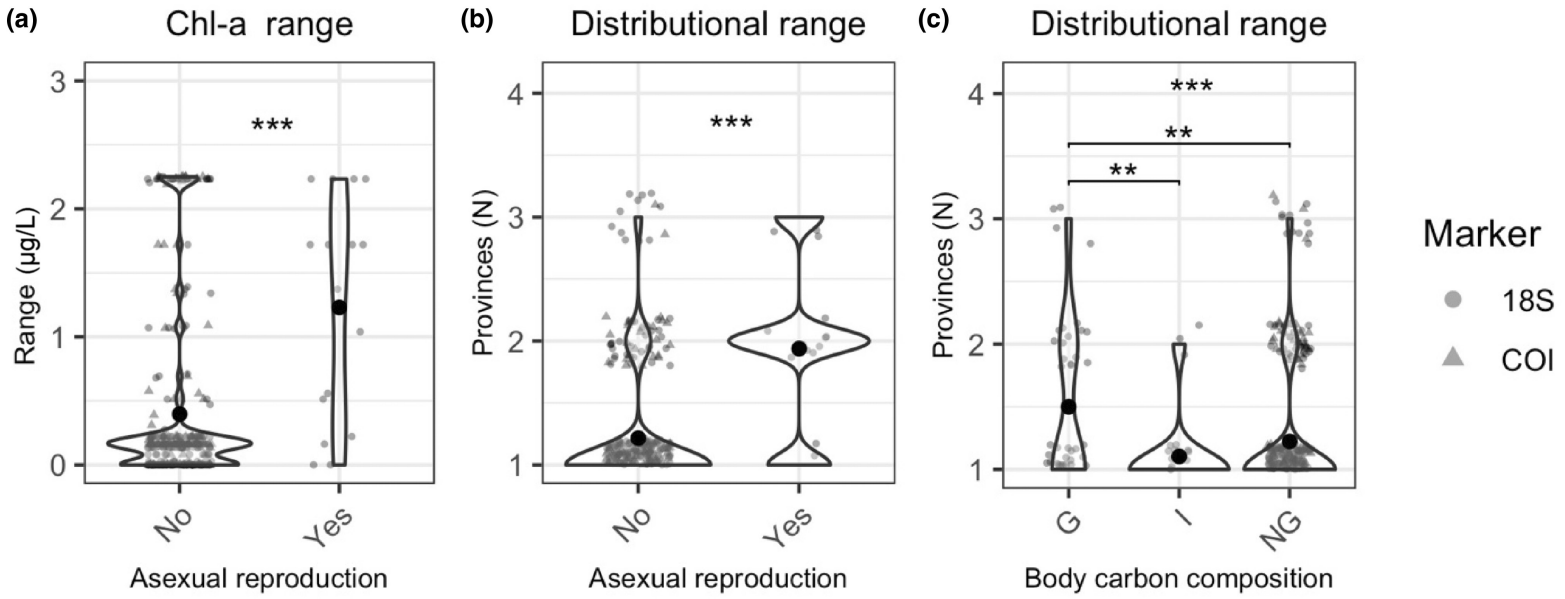
For epipelagic taxa, environmental and biogeographic ranges that differed among traits. (a) Chl‐a ranges for epipelagic taxa with and without asexual reproduction. (b) Number of biogeographic provinces across which taxa with and without asexual reproduction were distributed; (c) number of biogeographic provinces across which taxa with different body carbon compositions were distributed. Violin plots show density distribution with data points jittered within each categorical value. Data points are semi‐opaque; variability in color intensity reflects overlapping points. Circles denote taxa detected with 18S, and triangles denote taxa detected with COI. Global significance reflects the results of the Kruskal–Wallis test; pairwise comparisons are Bonferroni‐corrected Dunn's test results (ns = *p* > .05, *p < .05, **p < .01, ***p < .001).

To determine whether epipelagic and mesopelagic zooplankton with different ecological traits have different environmental and biogeographic ranges, we examined the environmental ranges across which taxa with different trait states for diet, feeding behavior, spawning strategy, presence of asexual reproduction, or DVM behavior were observed. Among epipelagic taxa, we found significant differences in the Chl‐*a* ranges and distributional ranges of taxa with and without the capacity for asexual reproduction, with asexually reproducing taxa found across wider Chl‐*a* ranges and more provinces (Chl‐*a*: χ^2^ = 13.804, 1 df, *p* < .001; number of provinces: *χ* ^2^ = 31.195, 1 df, *p* < .001) (Figure 10a,b). We also found significant differences in the number of provinces in which taxa with different body carbon compositions were observed, with gelatinous taxa more likely to be distributed across multiple provinces than either taxa with non‐gelatinous or intermediate compositions (*χ* ^2^ = 14.418, 2 df, *p* < .001) (Figure 10c).

**FIGURE 11:**
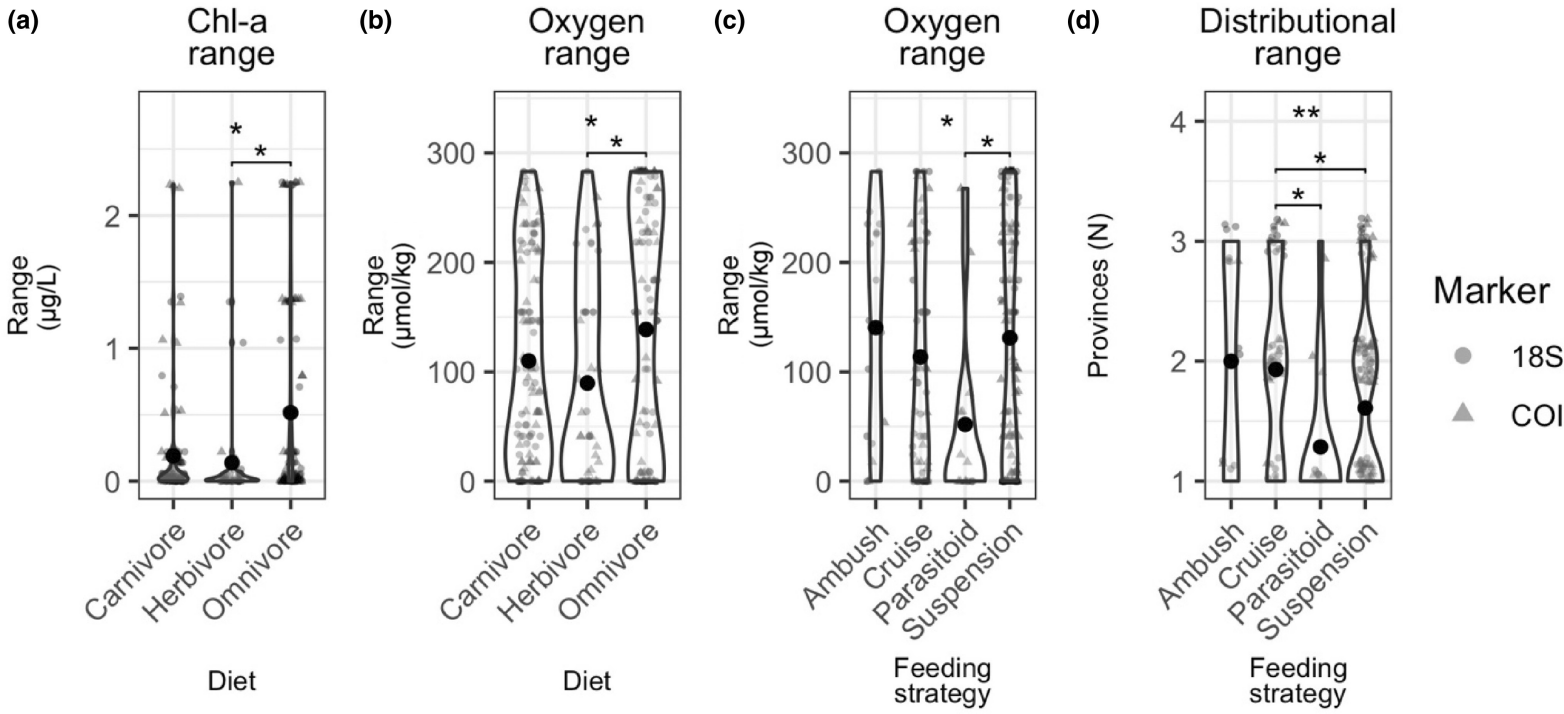
For mesopelagic taxa, the environmental and biogeographic ranges that differed among traits (a) Chl‐a ranges for mesopelagic taxa with different diets; (b) Oxygen ranges for mesopelagic taxa with different diets; (c) Oxygen ranges for mesopelagic taxa with different feeding strategies; and (d) Number of biogeographic provinces across which taxa with different feeding strategies were distributed. Violin plots show density distribution with data points jittered within each categorical value. Data points are semi‐opaque; variability in color intensity reflects overlapping points. Circles denote taxa detected with 18S, and triangles denote taxa detected with COI. Global significance reflects the results of the Kruskal–Wallis test; pairwise comparisons are Bonferroni‐corrected Dunn's test results (ns = *p* > .05, *p < .05, **p < .01, ***p < .001).

Among mesopelagic taxa, we found significant differences in the breadth of Chl‐*a* and dissolved oxygen ranges between taxa with different diets, with omnivores inhabiting wider ranges than herbivores (Chl‐*a*: *χ* ^2^ = 7.6532, 2 df, *p* < .05; oxygen: *χ* ^2^ = 6.8479, 2 df, *p* < .05) (Figure 11a,b). We also observed differences in the oxygen ranges and distributional extent of taxa with different feeding strategies, with suspension feeding taxa occurring across a wider range of oxygen concentrations than parasitoid feeding taxa (*χ* ^2^ = 8.2206, 3 df, *p* < .05) and cruise‐feeding taxa distributed across more provinces than either parasitoid taxa or suspension feeders (*χ* ^2^ = 13.613, 3 df, *p* < .01) (Figure 11c,d).

**FIGURE 12:**
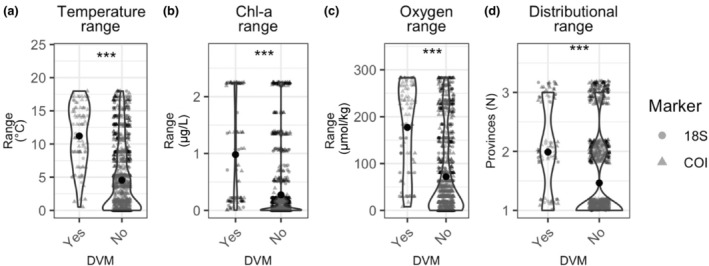
For taxa with and without diel vertical migration, (a) temperature ranges; (b) Chl‐a ranges; (c) oxygen ranges; and (d) the number of provinces each taxon was observed within. Violin plots show density distribution with data points jittered within each categorical value. Data points are semi‐opaque; variability in color intensity reflects overlapping points. Circles denote taxa detected with 18S, and triangles denote taxa detected with COI. Global significance reflects the results of the Kruskal–Wallis test (ns = *p* > .05, *p < .05, **p < .01, ***p < .001).

For taxa with and without DVM behavior, we tested for differences in environmental and biogeographic ranges of taxa across the full water column, as these taxa utilize both epipelagic and mesopelagic habitats. We found that taxa exhibiting DVM behavior were distributed across wider ranges of temperature, Chl‐*a*, and oxygen and were more likely to occur in multiple biogeographic provinces than taxa without DVM behavior (temperature: χ^2^ = 111.23, 1 df, *p* < .001; Chl‐*a*: χ^2^ = 105.11, 1 df, *p* < .001; oxygen: χ^2^ = 91.611, 1 df, *p* < .001; number of provinces: χ^2^ = 118.02, 1 df, *p* < .001) (Figure 12).

## 4 DISCUSSION

To explore potential drivers of biogeographic distributions, we examined epipelagic and mesopelagic environmental ranges, species traits, and distributions across the Subtropical, Transition, and Subarctic biogeochemical provinces in the North Pacific Ocean. Our results suggest that differences in traits among zooplankton species can lead to differential effects of physical dispersal and environmental selection on biogeographic distributions. We found that the temperature, dissolved oxygen, and Chl‐a ranges of zooplankton in the epipelagic and mesopelagic North Pacific reflected the environmental variability observed within each taxon's primary vertical habitat, with oxygen levels appearing as the main environmental driver in the mesopelagic zone. Within each vertical depth zone, larger environmental ranges were associated with broader biogeographic distributions, but overall, we observed larger distributional ranges of mesopelagic taxa compared to the ranges of epipelagic taxa. We discuss the potential implications of each of these results below.

### 4.1 Community composition

**FIGURE 13:**
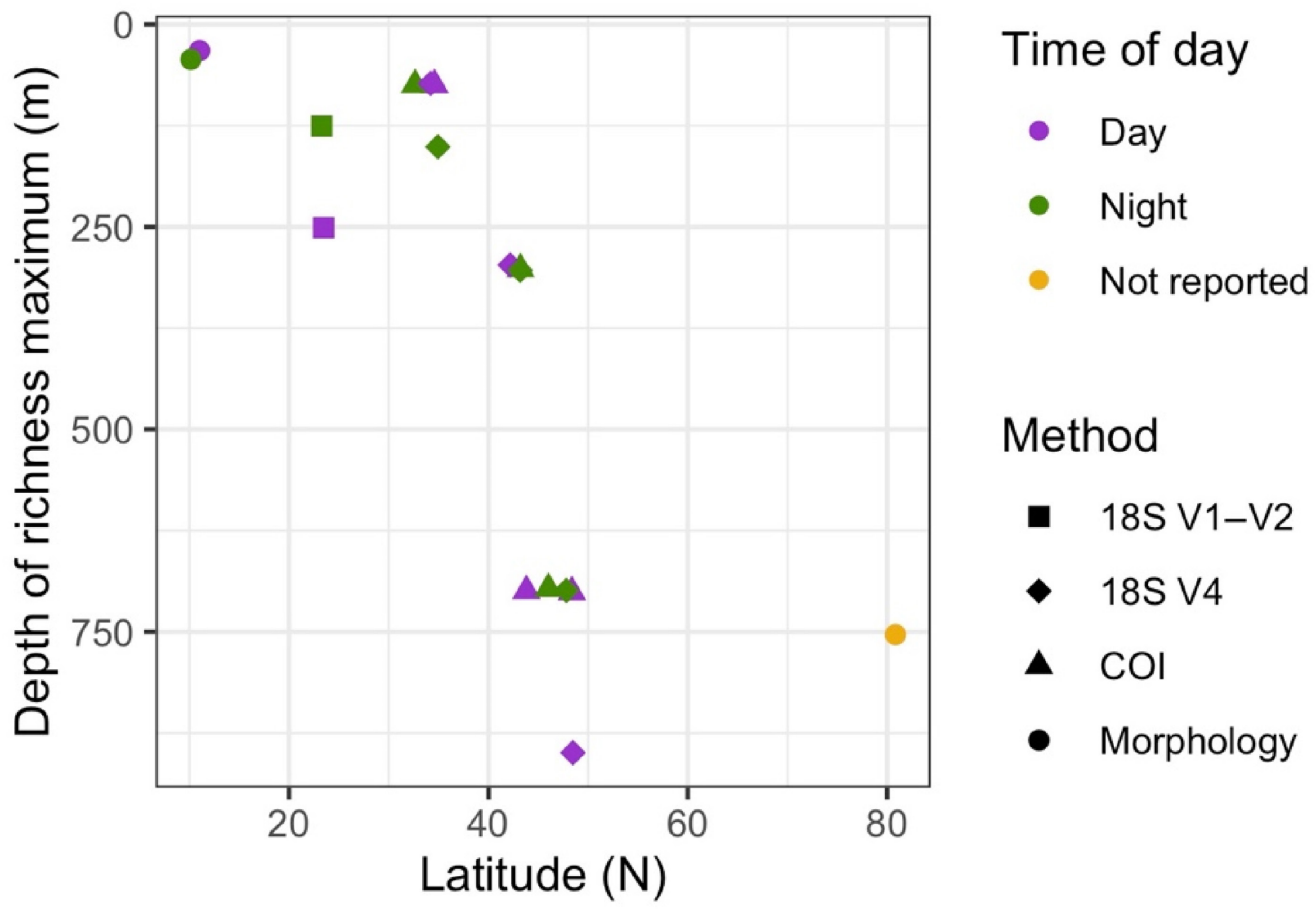
The depth of maximum zooplankton richness for sampling locations in the Pacific Basin, extending from the tropics to the Arctic. 18S V4 and COI data are from this study. 18S V1–V2 data are from Sommer et al., 2017. Morphological data are from Longhurst (1985) (tropics) and Kosobokova et al. (2011) (Arctic). Points are plotted at the midpoint for reported depth bins from each study.

Within both the epipelagic and mesopelagic zones, we observed the highest species richness in the Subtropical Province and decreasing richness at higher latitudes, with comparable decreases for the 18S and COI‐resolved zooplankton communities. Subtropical richness maxima and decreases in richness with increasing latitude are expected in pelagic ecosystems, although most previous analyses have focused on specific zooplankton groups and have often been restricted to the epipelagic (Angel, 1993; Brinton et al., 1999; Rombouts et al., 2009; van der Spoel et al., 1997). Our results reveal that latitudinal species richness in the mesopelagic parallels these previously described epipelagic trends. Additionally, vertical patterns of species richness varied among provinces, with mesopelagic richness maxima in the Transition and Subarctic Provinces but epipelagic richness maxima in the subtropics. Previous analyses of vertical gradients in zooplankton richness have found richness peaks at approximately 50 m in the Tropical Pacific (Longhurst, 1985), between 150 and 200 m in the Subtropical North Pacific (Sommer et al., 2017), and 500 and 1000 m in the Arctic basin (Kosobokova et al., 2011). Our data, combined with these previously published values, reveal a latitudinal trend of increasing depth of richness maxima with increasing latitude (Figure 13). The magnitude of richness cannot be compared among studies with different methodologies due to differences in taxonomic resolution, both between molecular and morphological approaches and among molecular markers. However, as long as a method was used consistently across the vertical range within a study, the depths of the richness maxima from independent studies can be compared. We caution against overinterpreting the slightly deeper richness maximum at lower subtropical latitudes due to variability in the longitudinal locations of these studies. Future studies should test the strength of this pattern.

Taxonomically, the zooplankton community was dominated by copepod reads, reflecting the importance of this major taxonomic group. The high proportions of Eucalanidae reads likely reflect preferential amplification of this family at the metabarcoding markers we used rather than true dominance of this group (Matthews et al., 2021). Previous analyses of zooplankton community composition have reported that copepods comprise 60–90% of the zooplankton community by both abundance and biomass in the Subtropical and Subarctic Pacific (Mackas & Tsuda, 1999; Steinberg et al., 2008). In the epipelagic Transition Province, the abundant malacostracan, hydrozoan, and thaliacean reads were primarily identified as euphausiids, siphonophores, and salps, respectively. This aligns with previous reports of increased euphausiid abundance in this region (Brodeur et al., 1999) and the association of thaliaceans with productive regions of ocean mixing (Ishak et al., 2022). Within the mesopelagic zone, the change from a community dominated by Metridinidae in the Subtropical Province to one dominated by Calanidae in the Subarctic Province agrees with previously described distributions. The genera *Gaussia*, *Metridia*, and *Pleuromamma* (all within the family Metridinidae) are associated with warmer subtropical regions (Soh et al., 1998), and mesopelagic Calanidae reads in the Subarctic Province reflect the tendency of high‐latitude herbivores to use the deep ocean as a refuge during diapause (Miller et al., 1984).

The trait composition of the full zooplankton community has not been previously well characterized for the depth ranges we examined. However, a few analyses are available for comparison. One study of the Subtropical Province used morphological identification of zooplankton communities in the upper 200 m (Ge et al., 2022). Similarly restricted to the upper ocean, copepod species distribution models in the upper 500 m have predicted that with increasing latitudes, copepods will shift toward larger body sizes, away from sac spawning, from carnivory to omnivory, and from ambush feeding to current and cruise feeding (Benedetti et al., 2022). Our body size observations do not align with these predicted latitudinal gradients. However, we used the maximum reported size for each species rather than in situ measurements of individual specimens, and there can be significant intraspecific variability in size (Kobari et al., 2003; Sasaki & Dam, 2020). Additionally, our analysis included the full zooplankton community rather than targeting copepod species, and it is possible that different taxonomic groups exhibit different body size responses to environmental drivers. The positive relationship between body size and depth for taxa smaller than 10 mm was stronger within specific zooplankton groups than across all zooplankton taxa combined. The apparent decreasing mean depth of occurrence for taxa between 10 and 100 mm was primarily driven by a few large (>100 mm) taxa; future studies could investigate whether zooplankton with body sizes >10 mm experience a decrease in visual predation risk (e.g., Ohman & Romagnan, 2016).

As predicted by Benedetti et al. (2022), we observed decreasing rates of brooding (sac spawning) with increasing latitude. Sac spawning was previously reported for about 50% of the zooplankton community (Ge et al., 2022), but we identified nearly 85% of reads as broadcast spawners in our study. Within copepods, broadcast spawning species may have large or small body sizes, while brooding species are moderately sized (Ohman & Townsend, 1998). The increased relative abundance of reads associated with broadcast spawning in our study more likely represents preferential amplification of taxonomic groups with brooding behavior rather than increased representation of higher biomass from these species.

Within the Subtropical Province, we observed similar proportions of feeding behaviors and dietary strategies as previously reported (Ge et al., 2022). Latitudinal increases in omnivory were also aligned with the prediction for copepods (Benedetti et al., 2022). However, we observed a peak in ambush feeding in the Transition Province rather than in the Subtropical Province, as predicted by Benedetti et al. (2022). Additionally, ambush feeding was more prevalent in the epipelagic than in the mesopelagic. This vertical difference in feeding behavior may reflect decreased physical mixing in the deep ocean, requiring animals to encounter prey items through motile cruise and suspension feeding.

Comparisons among trait‐based descriptions of the zooplankton community are limited by methodological differences as well as by minimal overlap in the study region. We calculated relative abundances of traits from metabarcoding reads, while previous studies have used morphology, whether from field‐collected samples (Ge et al., 2022) or from publicly available databases (Benedetti et al., 2022). Differences in relative abundances between metabarcoding reads and morphological counts may reflect methodological biases such as primer bias or gene copy number. Additionally, our metabarcoding reads can come from adults, juveniles, eggs, and debris. The traits assigned primarily describe the adult phase of the life history cycle (diet, feeding behavior, body composition) or are descriptive of the life history cycle as a whole (spawning strategy, potential for asexual reproduction). As such, our description of the trait composition of the zooplankton community reflects the hypothetical, adult‐dominated community. At any specific time period, the zooplankton community may be comprised of a significant proportion of juvenile stages, which may have different traits than we describe. However, for an autochthonous population of a zooplankton species to exist within a province, it must be able to complete its life history cycle. The traits of the adult stage, capable of reproduction, are a useful place to begin in describing the ecology of a community.

### 4.2 Biogeographic boundaries

We found no evidence for a biogeographic boundary between the mesopelagic Subarctic and Subtropical biogeochemical provinces. Across the three locations sampled, we observed greater community similarity in the mesopelagic zone than in the epipelagic zone, and we found broader distributional ranges for mesopelagic zooplankton. Decreased rates of endemism and greater community similarity could be created by increased connectivity, decreased environmental variability, or wider environmental tolerance windows for mesopelagic. However, connectivity through large‐scale ocean currents and physical mixing is muted in the mesopelagic compared to surface waters (Kawabe & Fujio, 2010; Reid et al., 1978; Scott et al., 2010). Greater similarity between mesopelagic communities than between epipelagic communities is paralleled by greater environmental similarity at mesopelagic depths than at epipelagic depths (Figures S5 and S6). Together with taxon‐specific environmental ranges that mirror the primary environmental variability found within each depth zone, greater community similarity and decreased rates of endemism suggest that midwater zooplankton community composition is more strongly influenced by environmental selection than by physical dispersal due to ocean mixing. Our results contrast with recent observations of decreased community similarity at depth and stronger effects of physical distance than of environmental distance on community similarity (Canals et al., 2023). However, our sampling locations comprise a strong latitudinal gradient in productivity, while the Pacific Ocean locations sampled by the Malaspina expedition were restricted to primarily equatorial and subtropical environments. The apparent conflicts between these two studies may indicate region‐specific patterns of similarity, reflecting varying strengths of physical connectivity and environmental dissimilarity.

To assess whether the North Pacific Transition Province is more strongly influenced by the northern Subarctic Province or the southern Subtropical Province, we examined Bray–Curtis community similarity and the absolute number of taxa shared among provinces. Within both the epipelagic and mesopelagic zones, we found higher proportions of shared taxa between samples from the Subtropical and Transition Provinces but greater Bray–Curtis similarities (incorporating relative abundance) between the Subarctic and Transition Provinces. The proportion of species shared between provinces and the overall community similarity between provinces will have different responses to physical dispersal, environmental similarity, and environmental tolerances of species. The number of species shared between provinces is a better measure of dispersal and physical connectivity, but can include expatriated individuals that are reproductively inactive. Similarity in community composition, incorporating the relative abundance of different species, is a better measure of the sum effects of dispersal, environment, and environmental tolerances. Previous latitudinal analyses of copepod community composition have found that the northern boundary of the Transition Province is a region of decreased physical mixing and muted community connectivity (Miyamoto et al., 2022), aligning with our finding that species within the Transition Province were more likely to be shared with the southern Subtropical Province than with the northern Subarctic Province. Conversely, the increased community‐level similarity we observed between the Transition and Subarctic Provinces mirrored the increased environmental similarity between these two provinces in temperature, salinity, Chl‐*a*, and dissolved oxygen, particularly within the epipelagic zone (Figures S5 and S6). The increased community‐level similarity between the Transition and Subarctic Provinces reflects higher abundances of taxa from the north, which may be better adapted to the cold, productive Transition Province. We discuss the potential role of environmental tolerance below.

### 4.3 Traits and habitat ranges

To investigate the potential effects of environmental tolerance on biogeographic ranges, we examined the temperature, Chl‐*a*, and oxygen ranges of epipelagic and mesopelagic taxa with varying biogeographic extent. The strongest environmental gradients in the pelagic ocean occur vertically, and gradients in temperature and Chl‐*a* are concentrated in the epipelagic zone (Miller, 2004), while strong oxygen gradients are more commonly found within the mesopelagic zone (Robinson et al., 2010). Epipelagic taxa were observed across broader temperature and Chl‐*a* ranges than mesopelagic taxa, and broader temperature and Chl‐*a* ranges were associated with larger biogeographic distributions. Similarly, mesopelagic taxa were observed across broader ranges of dissolved oxygen than epipelagic taxa, and mesopelagic taxa with broader dissolved oxygen ranges also exhibited larger biogeographic distributions.

Temperature is well established as a structuring variable for epipelagic zooplankton distributions (Beaugrand et al., 2014; Mackas et al., 2007) and community composition (Costello et al., 2017; Hirai et al., 2020). The importance of oxygen to mesopelagic community composition and species distributions is also well documented (Bertrand et al., 2011; Ekau et al., 2010; Wishner et al., 2020). The ranges of Chl‐*a* values observed for primarily mesopelagic taxa and the ranges of dissolved oxygen concentrations observed for epipelagic taxa indicate that these taxa are not restricted to their primary depth habitat. For both epipelagic and mesopelagic zooplankton, survival in and utilization of multiple vertical habitats appear to be associated with larger biogeographic ranges.

Among epipelagic taxa, we found significantly wider Chl‐*a* ranges and broader distributional ranges for zooplankton with the capacity for asexual reproduction. The mechanistic link between asexual reproduction and distributional range is unclear. Asexual reproduction may allow for reproduction even by expatriated individuals that are unlikely to encounter a mate, which could increase the effectiveness of physical dispersal processes. However, many sexually reproducing zooplankton taxa have strategies for sperm storage and reproduction by previously mated females in the absence of available mates (Hirche, 1997; Miller & Clemons, 1988; Næss & Nilssen, 1991). The larger biogeographic distributions of asexual taxa may reflect the mechanistic effects of other traits that covary with this trait. Future analyses should prioritize traits related to feeding and energy requirements, as Chl‐*a* ranges were also larger for asexually reproducing zooplankton.

Among mesopelagic taxa, we found the strongest differences in environmental and distributional ranges among taxa with different dietary strategies and feeding behaviors. Chl‐*a* and dissolved oxygen ranges were larger for omnivores than for herbivores. Dissolved oxygen ranges were also larger for suspension feeding taxa than for parasitoid taxa, but the widest biogeographic ranges were found in cruise‐feeding taxa. As far as we are aware, there are no previous observational studies of the environmental ranges associated with zooplankton feeding behaviors. Among studies of dietary strategies, there are conflicting results. Analyses of copepod taxa in the North Atlantic and Southern Oceans found no difference in the environmental niche breadth of omnivores and herbivores but found narrower environmental niches for carnivores (McGinty et al., 2018). The environmental ranges of herbivore and omnivore‐dominated cool‐water copepod assemblages in the western North Pacific are narrower than the ranges of more carnivorous warm‐water assemblages in the Subtropical North Pacific (Tang et al., 2022). While there are incongruent outcomes between previous studies and our results, there are also notable differences among studies. Differences in dietary definitions reflect differences in the taxonomic specificity and breadth of zooplankton taxa analyzed. We also note that both of these analyses focused on biogeographic distributions within the epipelagic zone, while our observations extend through the mesopelagic zone. The environmental ranges of zooplankton with different dietary strategies and feeding behaviors appear to vary among ecosystems and taxonomic groups.

Across all taxa, we found differences in the temperature, dissolved oxygen, Chl‐*a*, and distributional ranges of taxa with or without DVM behavior, with larger ranges for taxa with DVM. Due to the strong vertical gradients found in the pelagic ocean, DVM exposes taxa to multiple habitats and a wide range of environmental conditions. It is unclear whether the wider environmental ranges associated with DVM reflect transit through low‐oxygen layers or residence within hypoxic habitats. Similarly, DVM behavior can allow primarily herbivorous taxa to reside in habitats far below the euphotic zone for a portion of each day (Cohen & Forward, 2009). However, the larger distributional ranges we observed for taxa with DVM behavior indicate that this vertical habitat flexibility is associated with increased species ranges.

In addition to the traits that we explicitly tested, there are other characteristics that may be associated with specific depth zones or may influence the environmental ranges of zooplankton species. The variability we observed in the environmental and distributional ranges of taxonomic classes and copepod families may be used to identify additional traits that should be considered (Figures S7 and S8). Notably, generation time is highly variable among zooplankton: the generation time of crustaceans is typically on the order of 30–180 days, while generation times for thaliaceans in particular can be much shorter (Bone., 1998). The duration of developmental stages, total generation time, and flexibility in development can vary even within a species depending on temperature and food availability. Species inhabiting deeper depth zones may have longer lifespans, as animals typically exhibit lower metabolic rates at colder mesopelagic depths (Ikeda et al., 2007). Longer lifespans could result in larger distributional ranges due to increased dispersal potential over the lifetime of individual animals. As our metabarcoding methods do not resolve the life history stage of the animals collected or the reproductive potential of the individuals detected, we did not include generation time, development time prior to reproductive maturity, or capacity for indeterminate growth in our analysis. Future work could consider differences in dispersal potential and environmental tolerance due to variability in generation time and growth strategies.

### 4.4 Comparing and combining molecular markers

Multi‐locus comparisons can compensate for marker‐specific biases (Questel et al., 2021), and the use of 18S and COI in combination has been particularly useful for achieving broad taxonomic coverage while maintaining the capacity for differentiating among closely related species. Each marker provides a unique perspective on the community. Comparison between markers can be informative, and the merging of datasets from independent markers is not always justifiable. In the current study, vertical richness gradients differed slightly between the 18S and COI markers, and COI detected moderately higher richness than 18S in the mesopelagic zone, but there was no such difference between markers in the epipelagic. Malacostracans, ostracods, and gastropods were better detected at the COI marker; thaliaceans and most cyclopoid copepods were detected nearly exclusively by the 18S marker. Estimates of community diversity and composition are most accurately interpreted when presented independently for each marker, allowing for consideration of taxonomic biases and rates of molecular substitution.

In contrast, the combination of community composition data from multiple markers can increase confidence in some analyses when markers are thoughtfully combined in an appropriate manner. In our comparison of epipelagic and mesopelagic communities, the same biogeographic patterns were detected independently in the 18S‐ and COI‐resolved communities. In the absence of any significant differences between the two markers, we opted to combine the datasets for some analyses. Summing or averaging the abundances of each species detected in both datasets can enable comparison with non‐molecular methods (e.g., Meredith et al., 2021), but it requires high confidence in taxonomic assignments and can result in the loss of sequence variant differences. In the current study, markers were combined by treating each OTU or ASV as an independent observation of a species. This may result in double‐counting of taxa that were detected by both markers, but as multiple ASVs or OTUs within one marker are often assigned identical taxonomic strings and may or may not have similar distribution patterns, any bias introduced by double counting has less effect than taxonomic lumping.

The use of multiple independent methods in a single study is common in zooplankton ecology, with approaches ranging from the explicit comparison of methods (e.g., Hetherington et al., 2022; Whitmore et al., 2019) to the combination of multiple methods with diverging strengths in order to more fully constrain measurements (e.g., Bucklin et al., 2002). We suggest that studies employing multiple metabarcoding markers use both of these approaches. Comparison between markers can help constrain the scope of the zooplankton community resolved by each marker, and where community‐level patterns are independently detected by multiple markers, confidence in the results can be increased.

## 5 CONCLUSION

We found that the mesopelagic zooplankton community in the eastern North Pacific Ocean has greater similarity across biogeographic provinces than the epipelagic community. Mesopelagic taxa were observed across more biogeographic provinces than epipelagic taxa, and there was less differentiation among mesopelagic than among epipelagic zooplankton communities across the NPTZ. The temperature, dissolved oxygen, and Chl‐*a* ranges of epipelagic and mesopelagic taxa reflected the environmental variability observed within each taxon's primary vertical habitat. Larger environmental ranges were associated with broader biogeographic distributions, but the extent of environmental and distributional ranges varied among traits. These findings confirm the influence of physical dispersal regimes, environmental variability, and species traits in shaping zooplankton distributions throughout the upper 1000 m of the pelagic ocean. Physical dispersal can introduce zooplankton into new environments, but our results suggest that tolerance for those new environments may play a larger role in shaping the overall composition of the zooplankton community.

## AUTHOR CONTRIBUTIONS

**Stephanie A. Matthews:** Conceptualization (equal); data curation (equal); formal analysis (equal); funding acquisition (equal); investigation (equal); methodology (equal); project administration (equal); resources (equal); validation (equal); visualization (equal); writing – original draft (equal); writing – review and editing (equal). **Leocadio Blanco‐Bercial:** Funding acquisition (equal); investigation (equal); methodology (equal); resources (equal); writing – review and editing (equal).

## Supporting information


Figures S1–S4

Table S1

Table S2

Table S3

## Data Availability

Raw sequences are deposited in the NCBI nucleotide database under BioProject PRJNA954464. All code, CTD data, and sample metadata can be found at https://github.com/samatthews/NorthPacificMidwater.
