# Supplementary material for: Divergent patterns of zooplankton connectivity in the epipelagic and mesopelagic zones of the eastern North Pacific
Source: Ecol Evol. 2023 Nov 5;13(11):e10664. doi: 10.1002/ece3.10664 (PMC10625861; doi:10.1002/ece3.10664)
Supplement: Supplementary file 1 — Figures S1–S4 [file ECE3-13-e10664-s001.docx]

*Supplemental materials for:*

**Divergent patterns of zooplankton connectivity in the epipelagic and mesopelagic zones of the eastern North Pacific**

Stephanie A. Matthews, Leocadio Blanco-Bercial

**Table of contents:**

**Table S1:** Zooplankton specimens sequenced for inclusion in reference databases (separate file: TableS1.xlsx).

**Table S2A:** Summary statistics for sequencing depth at each marker (separate file: TableS2.xlsx).

**Table S2B:** Sample metadata and total sequencing depth at each marker for each biological sample after merging technical replicates (separate file: TableS2.xlsx).

**Table S2C:** Sequencing depth and number of zooplankton reads for all technical replicates of the COI marker (separate file: TableS2.xlsx).

**Table S2D:** Sequencing depth and number of zooplankton reads for all technical replicates of the 18S marker (separate file: TableS2.xlsx).

**Table S3:** Trait assignments and references for each unique taxonomic assignment (separate file: TableS3.xlsx).

**Figure S1:** Relationships between sequencing depth and richness for non-rarefied and rarefied data.

**Figure S2:** Richness patterns across depth, for rarefied data.

**Figure S3:** Taxon size across depth for taxonomic classes.

**Figure S4**: Environmental ranges of epipelagic and mesopelagic zooplankton taxa.

**Figure S5:** The range of environments observed within the epipelagic and mesopelagic zones for each province.

**Figure S6:** Principal component analysis of environmental similarity among MOCNESS samples.

**Figure S7**: Biogeographic extent of each taxonomic class and copepod families.

**Figure S8:** Environmental ranges for taxonomic classes and for copepod families.

**Supplemental References:** References for all literature cited in Table S3.


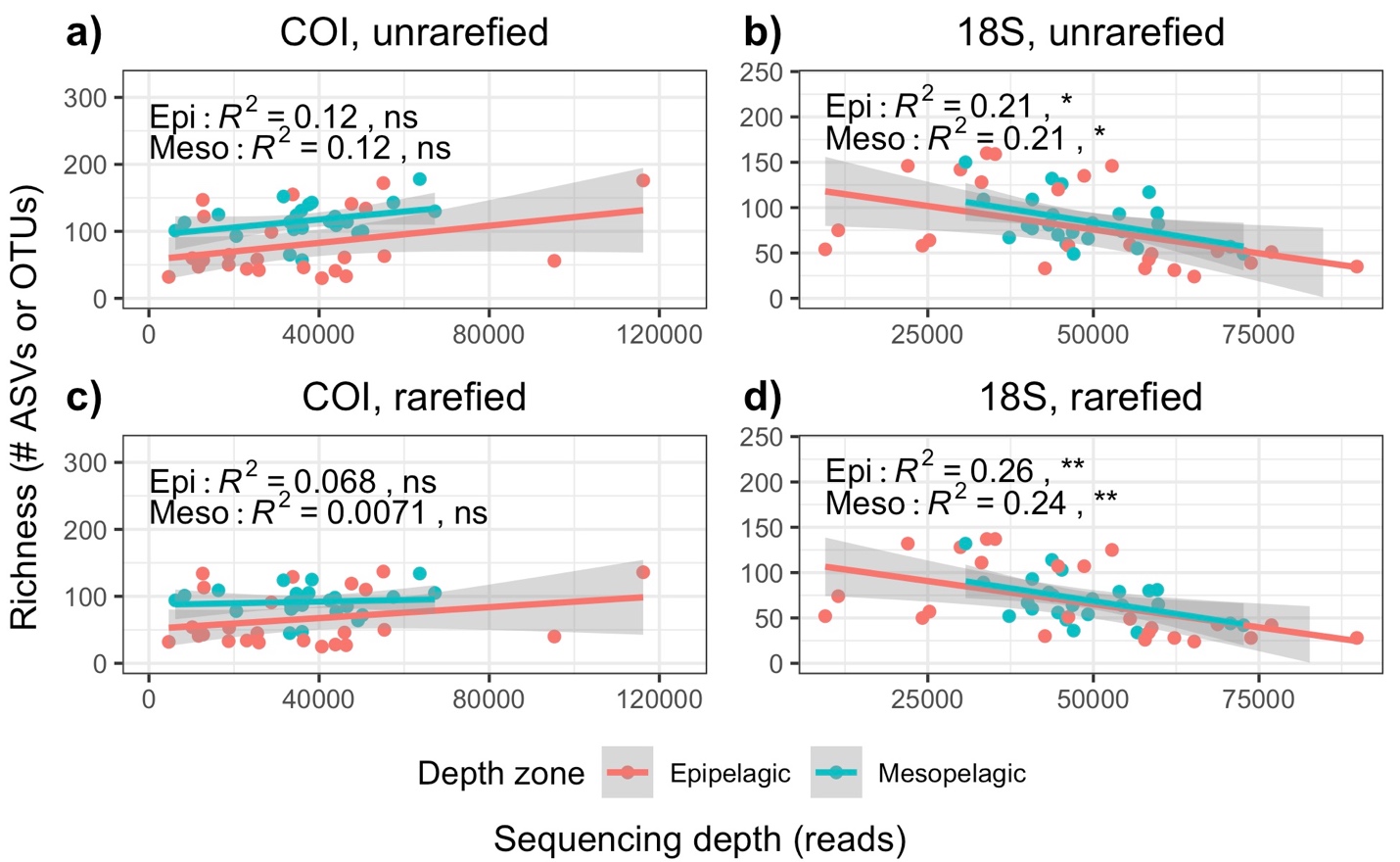


**Figure S1.** The Pearson correlation between sequencing depth prior to rarefaction and observed richness of a) and c): COI OTUs pre- and post- rarefaction; and b) and d): 18S ASVs pre- and post- rarefaction. Coral points and lines represent epipelagic samples (0-200m) and teal points and lines represent mesopelagic samples (200-1000m). Rarefied richness in (c) and (d) are plotted against the original sequencing depth, as samples have identical numbers of reads post-rarefaction. Shading surrounding lines represents the 95% CI. (ns = p > 0.05, ∗ p < 0.05, ∗∗ p < 0.01, ∗∗∗ p < 0.001).


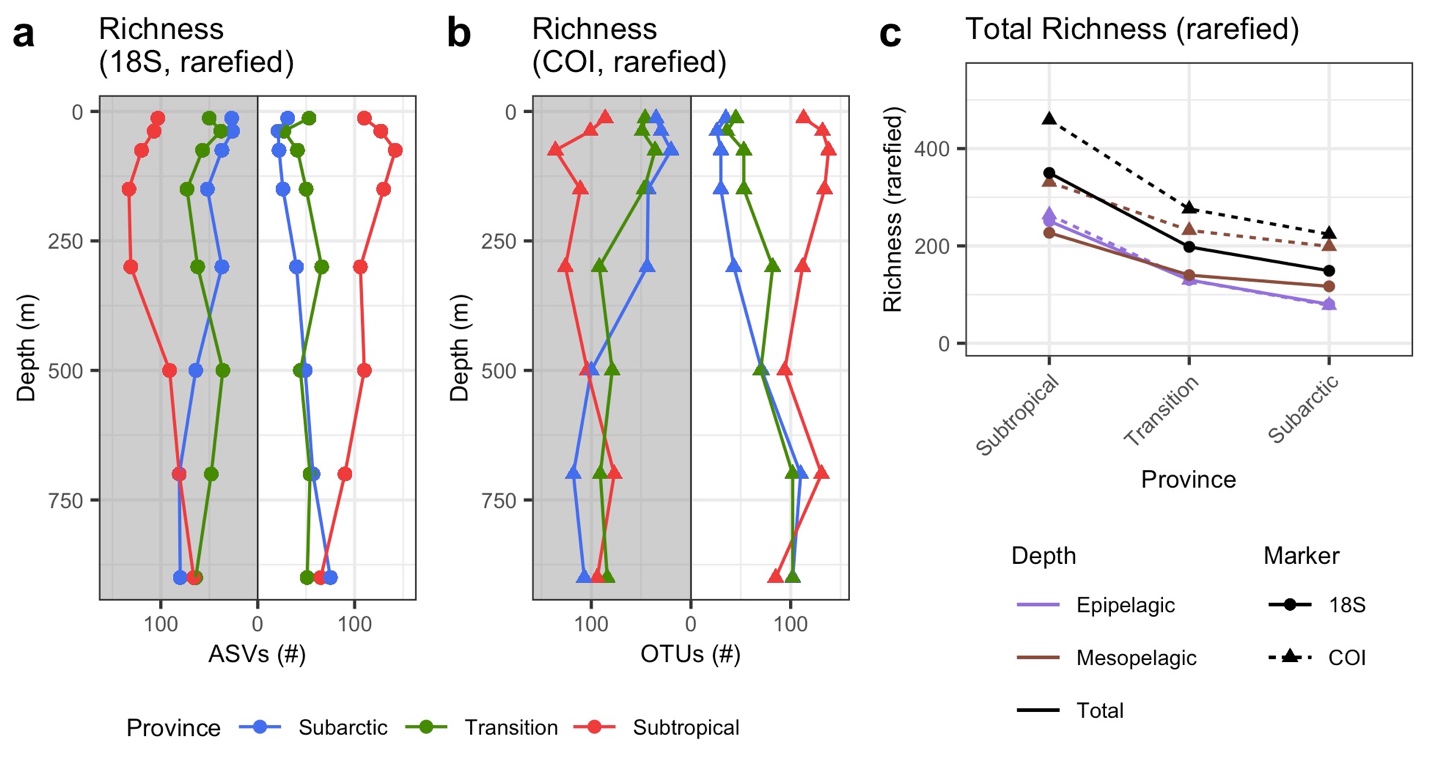


**Figure S2.** Taxonomic richness, calculated from rarefied datasets, across the three biogeographic provinces for (a) COI OTUs across depth (b) 18S ASVs across depth, and (c) total richness within the epipelagic zone, mesopelagic zone, and across the full 0-1000m sampled. For panels (a) and (b), points are plotted at the midpoint of each discrete zooplankton net range, with nighttime samples on the left (gray background) and daytime samples on the right (white background). For panel (c), colors indicate depth zones and line types denote marker. Richness is the total number of OTUs/ASVs for COI or 18S, respectively.


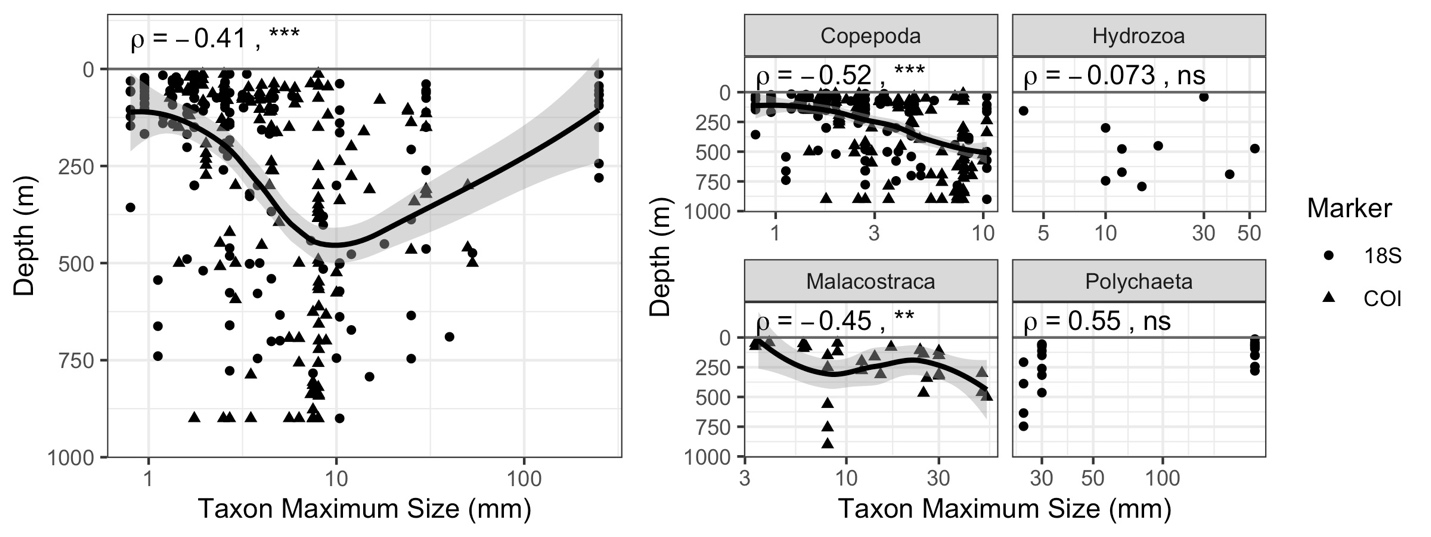


**Figure S3**. The Spearman rank correlation between the maximum reported size and the observed mean depth of occurrence for (a) all ASVs and OTUs together; and (b) ASVs and OTUs within taxonomic classes with at least 10 unique taxa. Circles denote taxa detected with 18S, triangles denote taxa detected with COI. Loess best fit lines are shown for significant relationships. Shading surrounding lines represents the 95% CI. (ns = p > 0.05, ∗ p < 0.05, ∗∗ p < 0.01, ∗∗∗ p < 0.001).


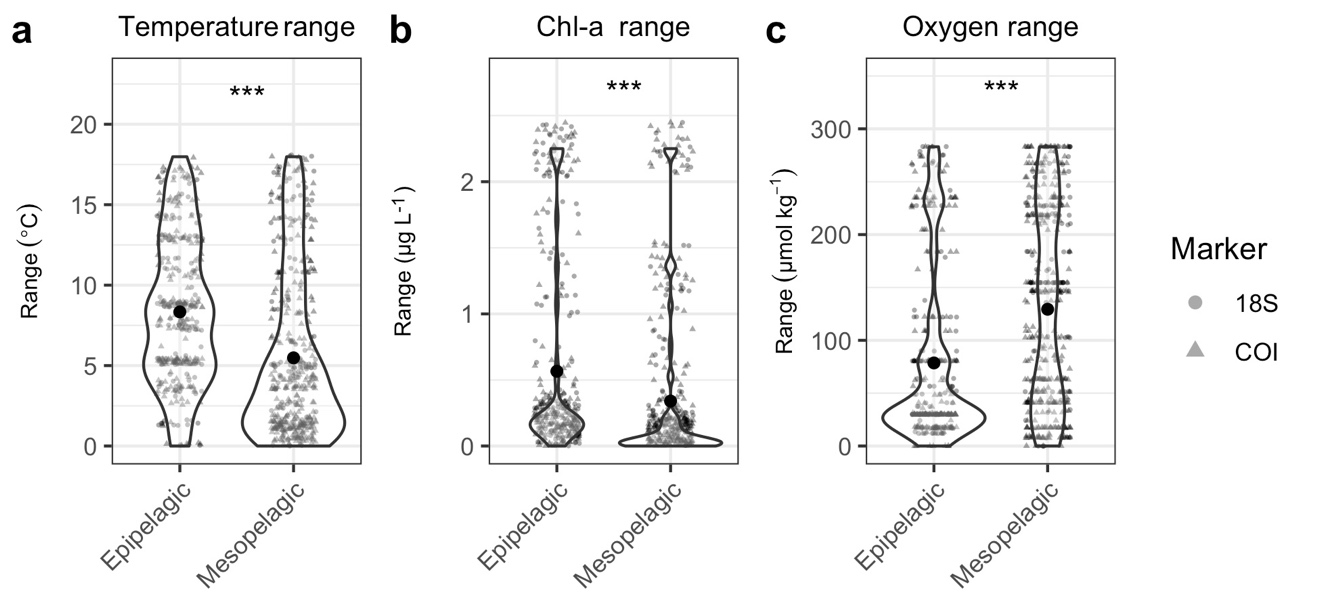


**Figure S4.** Epipelagic and mesopelagic OTUs and ASVs and the environmental ranges across which they were observed, for (a) temperature; (b) Chl-a fluorescence; and (c) dissolved oxygen. Violin plots showing the density distribution of points are overlaid on jittered data. Points are semi-opaque, variability in the intensity of color is due to overlapping data points. Circles denote taxa detected with 18S, triangles denote taxa detected with COI. (Kruskal-Wallis: ns = p > 0.05, ∗ p < 0.05, ∗∗ p < 0.01, ∗∗∗ p < 0.001).


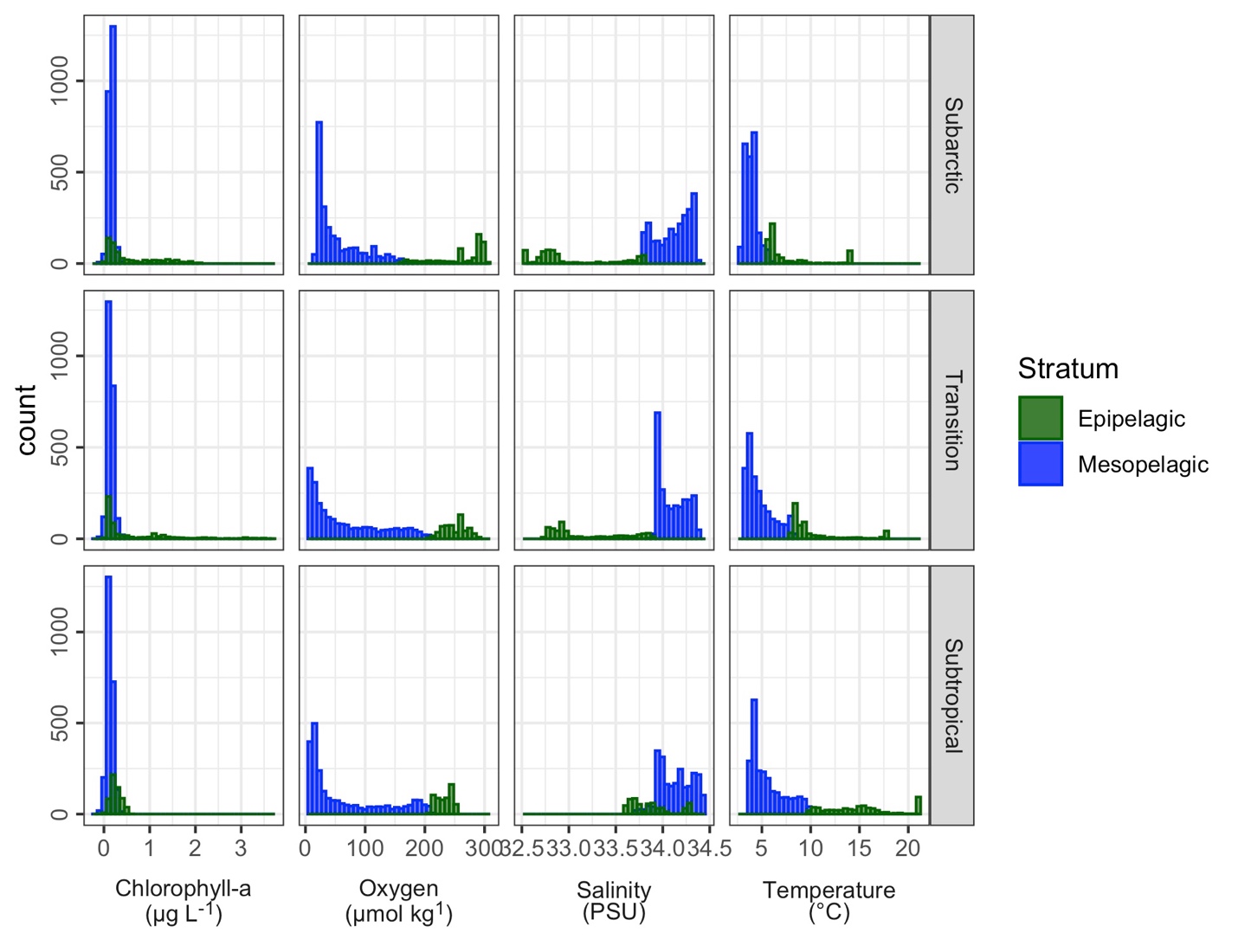


**Figure S5**. Histograms of the observed physical environment within the epipelagic zone (0-200m, green) and mesopelagic zone (200-1000m, blue) for temperature, salinity, Chl-a fluorescence, and dissolved oxygen (columns), within the Subarctic, Transition, and Subtropical biogeographic provinces (rows). Data are 1-meter averaged values from 0-1000m CTD casts.


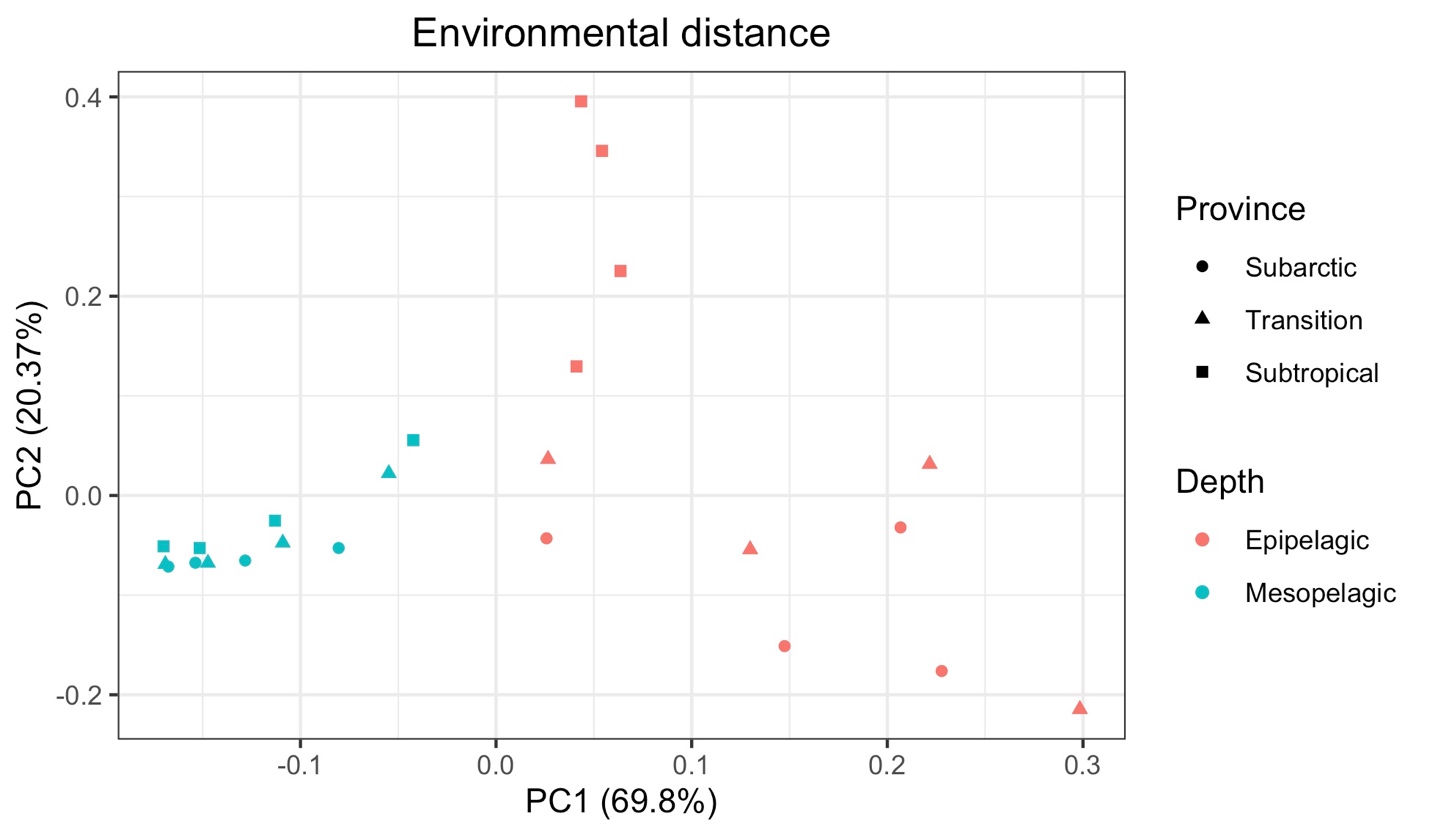


**Figure S6.** Principal component analysis of the environment observed within each net, including mean temperature, chl-a fluorescence, salinity, and dissolved oxygen. All environmental variables were scaled and centered prior to calculating distance. Shapes indicate different biogeographic provinces, and colors denote vertical depth zones.

*
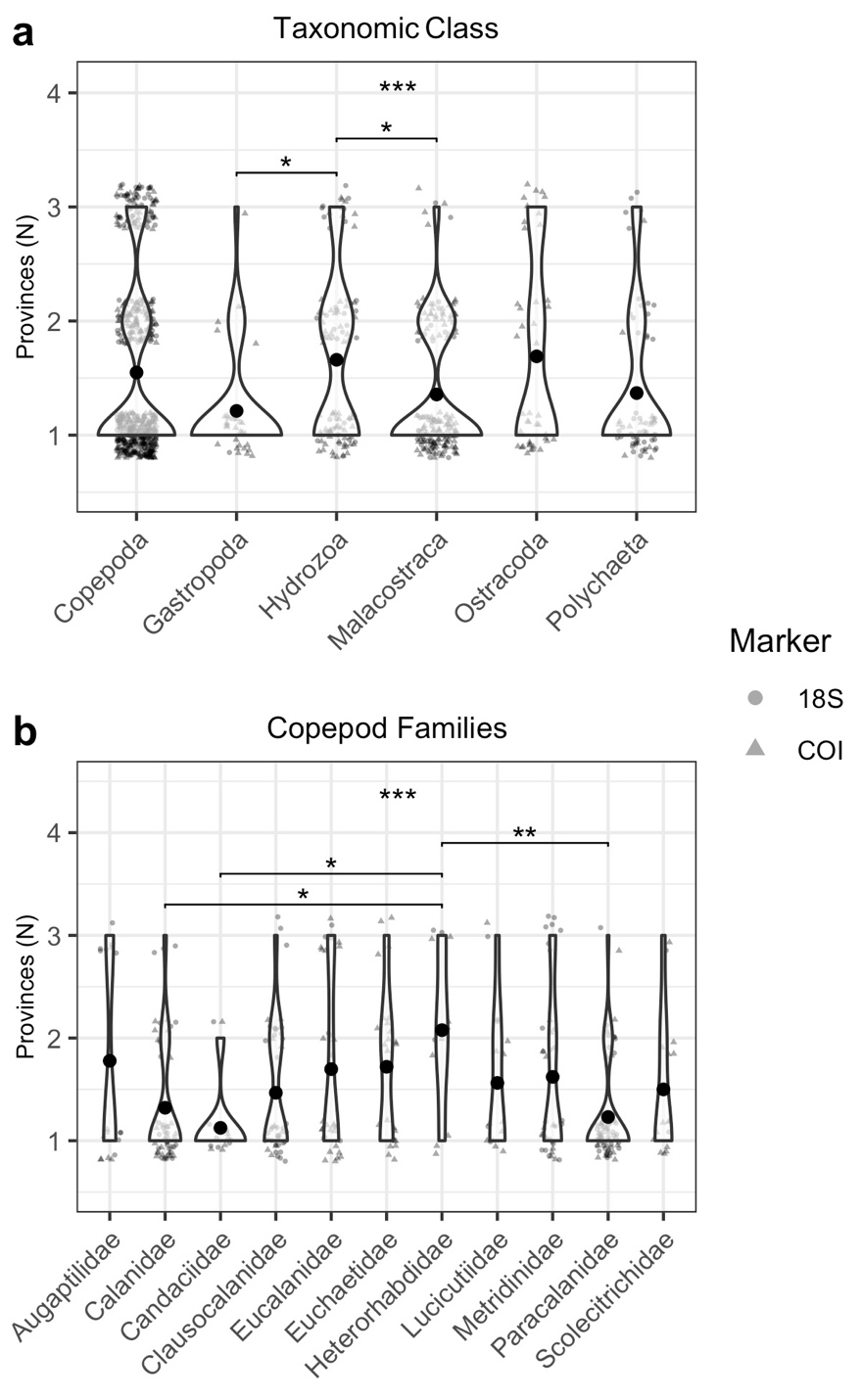
*

**Figure S7.** Number of provinces in which OTUs and ASVs were observed, divided by (a) taxonomic class; and (b) taxonomic family within the class Copepoda. Only taxonomic groups with at least 10 unique taxa are shown. Violin plots showing the density distribution of points are overlayed on data points jittered within each categorical value. Data points are semi-opaque, variability in color is due to overlapping data points. Circles denote taxa detected with 18S, triangles denote taxa detected with COI. (ns = p > 0.05, ∗ p < 0.05, ∗∗ p < 0.01, ∗∗∗ p < 0.001).


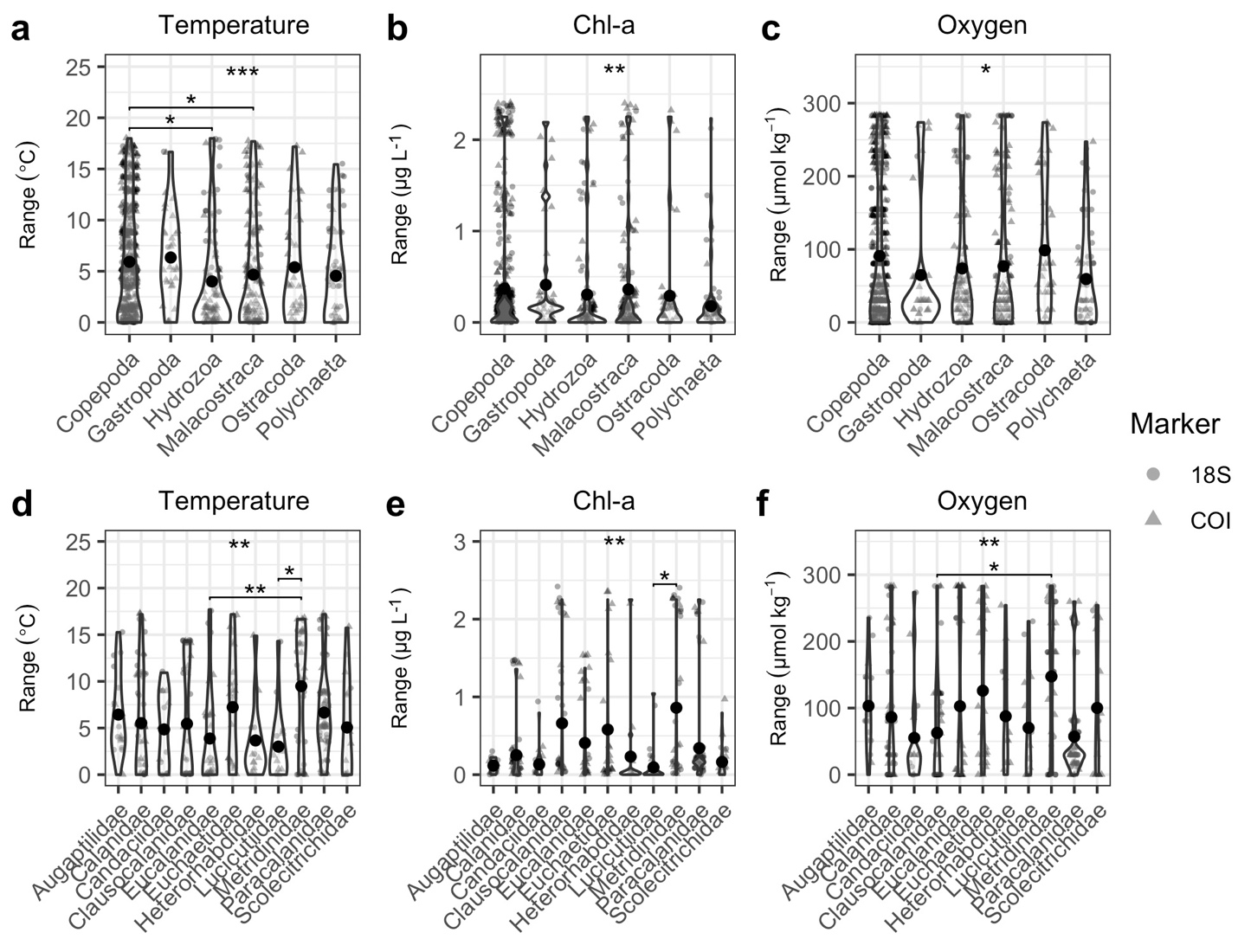


**Figure S8.** For each taxonomic group, OTUs and ASVs are plotted according to the environmental range across which they were observed, for (a) taxonomic class and temperature; (b) taxonomic class and Chl-a; (c) taxonomic class and oxygen; (d) copepod family and temperature; (e) copepod family and Chl-a; and (f) copepod family and oxygen. Violin plots showing the density distribution of points are overlayed on data points jittered within each categorical value. Data points are semi-opaque, variability in the intensity of color are due to overlapping data points. Circles denote taxa detected with 18S, triangles denote taxa detected with COI. (ns = p > 0.05, ∗ p < 0.05, ∗∗ p < 0.01, ∗∗∗ p < 0.001).

**Supplemental References**

Alvariño, A. 1992. Chaetognatha, p. 425–470. *In* Reproductive Biology of Invertebrates: V: Sexual Differentiation and Behaviour.

Alves-Júnior, F. D. A., E. D. S. Silva, M. D. S. L. C. De Araújo, I. Cardoso, A. Bertrand, and J. F. Souza-Filho. 2019. Taxonomy of deep-sea shrimps of the Superfamily Oplophoroidea Dana 1852 (Decapoda: Caridea) from Southwestern Atlantic. Zootaxa **4613**: 401. doi:[10.11646/zootaxa.4613.3.1](https://doi.org/10.11646/zootaxa.4613.3.1)

Angel, M. V., and A. W. G. John. 2017. Crustacea: Ostracoda, *In* Marine Plankton. Oxford University Press.

Arai, M. N. 1997. A Functional Biology of Scyphozoa, Springer Science & Business Media.

Astthorsson, O., and T. Brattegard. 2022. Útbreiðsla og líffræði agna (krabbadýr: Lophogastrida og Mysida) í hafinu við Ísland. Fjölrit Náttúrufræðistofnunar 114. doi:[10.33112/1027-832X.58](https://doi.org/10.33112/1027-832X.58)

Barnes, H., ed. 1984. Feeding in the Chaetognatha, *In* Oceanography and Marine Biology, An Annual Review, Volume 22. CRC Press.

Benedetti, F., S. Gasparini, and S.-D. Ayata. 2016. Identifying copepod functional groups from species functional traits. Journal of Plankton Research **38**: 159–166. doi:[10.1093/plankt/fbv096](https://doi.org/10.1093/plankt/fbv096)

Berkeley, E., and C. Berkeley. 1953. Micronereis nanaimoensis sp. n.: with some Notes on its Life-History. J. Fish. Res. Bd. Can. **10**: 85–95. doi:[10.1139/f53-007](https://doi.org/10.1139/f53-007)

Berkes, F. 1975. Some Aspects of Feeding Mechanisms of Euphausiid Crustaceans. Crustaceana **29**: 266–270.

Berning, M. R. 2014. The Feeding Ecology of Two Species of Holopelagic Munnopsid Isopods from the North Pacific (Acanthamunnopsis milleri and Munneurycope murrayi) Using SEM Analysis. Florida State University.

Bibermair, J., A. N. Ostrovsky, A. Wanninger, and T. Schwaha. 2021. Reproductive biology, embryonic development and matrotrophy in the phylactolaemate bryozoan Plumatella casmiana. Org Divers Evol **21**: 467–490. doi:[10.1007/s13127-021-00497-w](https://doi.org/10.1007/s13127-021-00497-w)

Blake, J. A. 1979. Proceedings of the Biological Society of Washington. Proceedings of the Biological Society of Washington **92**: 606–617.

Blaxter, J. H. S., F. S. Russell, and M. Yonge, eds. 1980. The Gut, Food and Feeding, p. 81–97. *In* Academic Press.

Boero, F., J. Bouillon, and S. Piraino. 1992. On the origins and evolution of hydromedusan life cycles (Cnidaria, Hydrozoa). In R. Dallai (ed.) Sex origin and evolution. **Selected Symposia and Monograph UZI 6. Mucchi**: 59–68.

Boltovskoy, D. 1999. South Atlantic Zooplankton, Backhuys.

Bone. 1998. The Biology of Pelagic Tunicates, Oxford University Press.

Bonifazi, A., E. Mancini, and D. Ventura. 2018. First record of the invasive and cryptogenic species Jassa slatteryi Conlan, 1990 (Crustacea: Amphipoda) in Italian coastal waters. Journal of Sea Research **136**: 37–41. doi:[10.1016/j.seares.2018.03.005](https://doi.org/10.1016/j.seares.2018.03.005)

Bottger-Schnack, R., J. Lenz, and H. Weikert. 2004. Are taxonomic details of relevance to ecologists? An example from oncaeid microcopepods of the Red Sea. Marine Biology **144**: 1127–1140. doi:[10.1007/s00227-003-1272-8](https://doi.org/10.1007/s00227-003-1272-8)

Bowman, T. E. 1985. The correct identity of the pelagic amphipod Primno macropa, with a diagnosis of Primno abyssalis. PROC. BIOL. SOC. WASH **98**: 121–126.

Bradford-Grieve, J. M., L. Blanco-Bercial, and G. A. Boxshall. 2017. Revision of Family Megacalanidae (Copepoda: Calanoida). Zootaxa **4229**: 1. doi:[10.11646/zootaxa.4229.1.1](https://doi.org/10.11646/zootaxa.4229.1.1)

Brinckmann-Voss, A. 2000. The hydroid and medusa of Sarsia bella sp. nov.(Hydrozoa, Anthoathecatae, Corynidae), with a correction of the life cycle of Polyorchis penicillatus (Eschscholtz). Scientia Marina **64**: 189–195.

Brinton, E., M. D. Ohman, A. W. Townsend, M. D. Knight, and A. L. Bridgeman. 1999. Euphausiids of the World Ocean - World Biodiversity Database CD-ROM Series.

Brinton, E., and A. W. Townsend. 1980. Euphausiids in the Gulf of California—the 1957 cruises. CalCOFI 21. 21.

Brun, P., M. R. Payne, and T. Kiørboe. 2017. A trait database for marine copepods. Earth System Science Data **9**: 99–113. doi:[10.1594/PANGAEA.862968](https://doi.org/10.1594/PANGAEA.862968)

Burnette, A. B., T. H. Struck, and K. M. Halanych. 2005. Holopelagic Poeobius meseres (“Poeobiidae,” Annelida) Is Derived From Benthic Flabelligerid Worms. The Biological Bulletin **208**: 213–220. doi:[10.2307/3593153](https://doi.org/10.2307/3593153)

Buskey, E. J. 1998. Energetic costs of swarming behavior for the copepod Dioithona oculata. Marine Biology **130**: 425–431. doi:[10.1007/s002270050263](https://doi.org/10.1007/s002270050263)

Casanova, J.-P. 1991. Chaetognaths from the *Alvin* dives on the Seamount Volcano 7 (east tropical Pacific). J Plankton Res **13**: 539–548. doi:[10.1093/plankt/13.3.539](https://doi.org/10.1093/plankt/13.3.539)

Chavtur, V. G., and D. Keyser. 2015. Morphology and distribution of pelagic ostracods of the genus Boroecia (Ostracoda: Halocyprididae) in the Central Arctic. Zootaxa **4013**: 151. doi:[10.11646/zootaxa.4013.2.1](https://doi.org/10.11646/zootaxa.4013.2.1)

Childress, J. J., and M. H. Price. 1983. Growth rate of the bathypelagic crustacean Gnathophausia ingens (Mysidacea: Lophogastridae). Mar. Biol. **76**: 165–177. doi:[10.1007/BF00392733](https://doi.org/10.1007/BF00392733)

Choi, H., T. W. Jung, and S. Yoon. 2015. A New Record of Glycerid Polychaete, Glycera fallax (Polychaeta: Glyceridae) from Korea. Korean Journal of Environmental Biology **33**: 274–278. doi:[10.11626/KJEB.2015.33.3.274](https://doi.org/10.11626/KJEB.2015.33.3.274)

Cohen, A. C., and J. G. Morin. 1990. Patterns of Reproduction in Ostracodes: A Review. Journal of Crustacean Biology **10**: 184–211. doi:[10.2307/1548480](https://doi.org/10.2307/1548480)

Corkett, C. J., and I. A. McLaren. 1979. The Biology of Pseudocalanus, p. 1–231. *In* Advances in Marine Biology. Elsevier.

Cowles, D. 2014. Salish Sea Invertebrates.

Cuzin-Roudy, J. 2010. Reproduction in Northern krill (Meganyctiphanes norvegica Sars). Adv Mar Biol **57**: 199–230. doi:[10.1016/B978-0-12-381308-4.00007-8](https://doi.org/10.1016/B978-0-12-381308-4.00007-8)

Dales, K. P. 1957. Pelagic Polychaetes of the Pacific Ocean. 73.

Daly, M., D. G. Fautin, and V. A. Cappola. 2003. Systematics of the Hexacorallia (Cnidaria: Anthozoa). Zoological Journal of the Linnean Society **139**: 419–437. doi:[10.1046/j.1096-3642.2003.00084.x](https://doi.org/10.1046/j.1096-3642.2003.00084.x)

Davies, C. H., and A. S. Skotwinski. 2012. Evadne nordmanni - Zooplankton.

Deevey, G. 1968. Pelagic ostracods of the Sargasso Sea off Bermuda: description of species seasonal and vertical distribution, Yale University.

Eckelbarger, K. J., and S. A. Rice. 1988. Ultrastructure of oogenesis in the holopelagic polychaetes Rhynchonerella angelini and Alciopa reynaudii (Polychaeta: Alciopidae). Marine Biology **98**: 427–439. doi:[10.1007/BF00391119](https://doi.org/10.1007/BF00391119)

Falk-Petersen, S., P. Mayzaud, G. Kattner, and J. R. Sargent. 2009. Lipids and life strategy of Arctic *Calanus*. Marine Biology Research **5**: 18–39. doi:[10.1080/17451000802512267](https://doi.org/10.1080/17451000802512267)

Fauchald, K., and P. A. Jumars. 1979. The diet of worms: a study of polychaete feeding guilds. Oceanography and marine Biology annual review **17**: 193–284.

Ferrari, F. D., and J. W. Ambler. 1992. Nauplii and copepodids of the Cyclopoid copepod Dioithona oculata (Farran, 1913) (Oithonidae) from a mangrove cay in Belize. Proceedings of the Biological Society of Washington **105**: 275–298.

Gibbons, M. J., L. A. Janson, A. Ismail, and T. Samaai. 2010. Life cycle strategy, species richness and distribution in marine Hydrozoa (Cnidaria: Medusozoa). Journal of Biogeography **37**: 441–448. doi:[10.1111/j.1365-2699.2009.02226.x](https://doi.org/10.1111/j.1365-2699.2009.02226.x)

Gómez-Gutiérrez, J. 2002. Hatching mechanism and delayed hatching of the eggs of three broadcast spawning euphausiid species under laboratory conditions. Journal of Plankton Research **24**: 1265–1276. doi:[10.1093/plankt/24.12.1265](https://doi.org/10.1093/plankt/24.12.1265)

Gómez-Gutiérrez, J. 2003. Hatching mechanism and accelerated hatching of the eggs of a sac-spawning euphausiid Nematoscelis difficilis. Journal of Plankton Research **25**: 1397–1411. doi:[10.1093/plankt/fbg095](https://doi.org/10.1093/plankt/fbg095)

Gómez-Gutiérrez, J., W. T. Peterson, and C. B. Miller. 2010. Embryo biometry of three broadcast spawning euphausiid species applied to identify cross-shelf and seasonal spawning patterns along the Oregon coast. Journal of Plankton Research **32**: 739–760. doi:[10.1093/plankt/fbq028](https://doi.org/10.1093/plankt/fbq028)

Güreşen, S. O., and O. Gönülal. 2018. New records of pelagic fauna from the Turkish waters. Turk J Zool 4.

Haddock, S. H. D. 2007. Comparative feeding behavior of planktonic ctenophores. Integrative and Comparative Biology **47**: 847–853. doi:[10.1093/icb/icm088](https://doi.org/10.1093/icb/icm088)

Hetherington, E. D., A. Damian‐Serrano, S. H. D. Haddock, C. W. Dunn, and C. A. Choy. 2022. Integrating siphonophores into marine food‐web ecology. Limnol Oceanogr Letters **7**: 81–95. doi:[10.1002/lol2.10235](https://doi.org/10.1002/lol2.10235)

Hiller-Adams, P., and J. J. Childress. 1983. Effects of feeding, feeding history, and food deprivation on respiration and excretion rates of the bathypelagic mysid Gnathophausia ingens. The Biological Bulletin **165**: 182–196. doi:[10.2307/1541363](https://doi.org/10.2307/1541363)

Hwang, D.-S., K.-W. Lee, J. Han, H. G. Park, J. Lee, Y.-M. Lee, and J.-S. Lee. 2010. Molecular characterization and expression of vitellogenin (Vg) genes from the cyclopoid copepod, Paracyclopina nana exposed to heavy metals. Comparative Biochemistry and Physiology Part C: Toxicology & Pharmacology **151**: 360–368. doi:[10.1016/j.cbpc.2009.12.010](https://doi.org/10.1016/j.cbpc.2009.12.010)

Ikeda, T., F. Sano, A. Yamaguchi, and T. Matsuishi. 2007. RNA:DNA ratios of calanoid copepods from the epipelagic through abyssopelagic zones of the North Pacific Ocean. Aquat. Biol. **1**: 99–108. doi:[10.3354/ab00011](https://doi.org/10.3354/ab00011)

Inatsuchi, A., S. Yamato, and Y. Yusa. 2010. Effects of temperature and food availability on growth and reproduction in the neustonic pedunculate barnacle Lepas anserifera. Mar Biol **157**: 899–905. doi:[10.1007/s00227-009-1373-0](https://doi.org/10.1007/s00227-009-1373-0)

Janssen, A. W. 2005. Development of Cuvierinidae (Mollusca, Euthecosomata, Cavolinioidea) during the Cainozoic: a non-cladistic approach with a re-interpretation of Recent taxa. Basteria **69**: 25–72.

Jepsen, P. M., H. van Someren Gréve, K. N. Jørgensen, K. G. W. Kjær, and B. W. Hansen. 2021. Evaluation of high-density tank cultivation of the live-feed cyclopoid copepod Apocyclops royi (Lindberg 1940). Aquaculture **533**: 736125. doi:[10.1016/j.aquaculture.2020.736125](https://doi.org/10.1016/j.aquaculture.2020.736125)

Jo, S., C. Ma, H. Suh, and S. Y. Hong. 1998. Mysidacea (Crustacea) from the Korea Strait and its adjacent waters. Korean Journal of Biological Sciences **2**: 33–47. doi:[10.1080/12265071.1998.9647388](https://doi.org/10.1080/12265071.1998.9647388)

Jo, Y. W. 1990. Oedicerotid Amphipoda (Crustacea) from shallow waters of Korea. Beaufortia **39**: 155–200.

Johnson, W. S., M. Stevens, and L. Watling. 2001. Reproduction and development of marine peracaridans, p. 105–260. *In* Advances in Marine Biology. Elsevier.

Jones, D. A., M. Kumlu, L. Le Vay, and D. J. Fletcher. 1997. The digestive physiology of herbivorous, omnivorous and carnivorous crustacean larvae: a review. Aquaculture **155**: 285–295. doi:[10.1016/S0044-8486(97)00129-4](https://doi.org/10.1016/S0044-8486(97)00129-4)

Kasatkina, A. P. 2003. Finding of a New Species of the Genus Caecosagitta in the Southwestern Bering Sea and the Revision of Sagitta macrocephala s. lato (Chaetognatha). **29**: 8.

Katechakis, A., and H. Stibor. 2004. Feeding selectivities of the marine cladocerans Penilia avirostris, Podon intermedius and Evadne nordmanni. Marine Biology **145**: 529–539. doi:[10.1007/s00227-004-1347-1](https://doi.org/10.1007/s00227-004-1347-1)

Keil, K. E., and K. J. Osborn. Associations between hyperiid amphipods and gelatinous zooplankton.

Kensley, B. F. 1971. The genus Gennadas in the waters around southern Africa. Annals of the South African Museum.

Kikuchi, T. 1991. Meso-or Bathypelagic Shrimps of the Family Oplophoridae (Crustacea: Decapoda) from the Western North Pacific: Part 1. Genus Meningodora Smith, 1882.

Kinsey, S. T., and T. L. Hopkins. 1994. Trophic strategies of euphausiids in a low-latitude ecosystem. Marine Biology **118**: 651–661. doi:[10.1007/BF00347513](https://doi.org/10.1007/BF00347513)

Kiørboe, T. 2013. Zooplankton body composition. Limnology and Oceanography **58**: 1843–1850. doi:[10.4319/lo.2013.58.5.1843](https://doi.org/10.4319/lo.2013.58.5.1843)

Komai, T., and T.-Y. Chan. 2010. A new genus and two new species of alvinocaridid shrimps (Crustacea: Decapoda: Caridea) from a hydrothermal vent field off northeastern Taiwan. Zootaxa **2372**: 15–32. doi:[10.11646/zootaxa.2372.1.3](https://doi.org/10.11646/zootaxa.2372.1.3)

Korovchinsky, N. M. 2000. Redescription of Diaphanosoma dubium Manuilova, 1964 (Branchiopoda: Ctenopoda: Sididae), and description of a new, related species. 20.

Kosobokova, K. N., and R. R. Hopcroft. 2021. Population structure, vertical distribution and fecundity of Eukrohnia hamata (Chaetognatha) in the Arctic Ocean during summer. Deep Sea Research Part I: Oceanographic Research Papers **169**: 103454. doi:[10.1016/j.dsr.2020.103454](https://doi.org/10.1016/j.dsr.2020.103454)

Kramp. 1968. Hydromedusae of the Pacific and Indian Oceans: Sections 2 and 3, Brill.

Krygier, E. E., and R. A. Wasmer. 1976. Description and biology of a new species of pelagic penaeid shrimp, Bentheogennema burkenroadi. Reference **76**: 44.

Lalli, C. M., and R. W. Gilmer. 1989. Pelagic Snails: The Biology of Holoplanktonic Gastropod Mollusks, Stanford University Press.

Larson, R. J., C. E. Mills, and G. R. Harbison. 1989. *In Situ* Foraging and Feeding Behaviour of Narcomedusae (Cnidaria: Hydrozoa). J. Mar. Biol. Ass. **69**: 785–794. doi:[10.1017/S002531540003215X](https://doi.org/10.1017/S002531540003215X)

Laval, P. 1980. Hyperiid amphipods as crustacean parasitoids associated with gelatinous zooplankton. Oceanography and Marine Biology Annual Review **18**: 11–56.

Lively, C. M., and S. G. Johnson. 1994. Brooding and the evolution of parthenogenesis: strategy models and evidence from aquatic invertebrates. Proceedings of the Royal Society of London. Series B: Biological Sciences **256**: 89–95.

Mackie, G. O., P. R. Pugh, and J. E. Purcell. 1988. Siphonophore Biology, p. 97–262. *In* Advances in Marine Biology. Elsevier.

Mapstone, G. 2015. Correction: Global Diversity and Review of Siphonophorae (Cnidaria: Hydrozoa). PLoS ONE **10**: e0118381. doi:[10.1371/journal.pone.0118381](https://doi.org/10.1371/journal.pone.0118381)

Markhaseva, E. L., and J. Renz. 2011. Two new Byrathis species (Copepoda: Calanoida) from the deep South Atlantic and Southern Ocean and first description of an adult male. Zootaxa **2889**: 49–68. doi:[10.11646/zootaxa.2889.1.3](https://doi.org/10.11646/zootaxa.2889.1.3)

Martindale, M. Q., and J. Q. Henry. 2015. Ctenophora, p. 179–201. *In* A. Wanninger [ed.], Evolutionary Developmental Biology of Invertebrates 1: Introduction, Non-Bilateria, Acoelomorpha, Xenoturbellida, Chaetognatha. Springer.

Mauchline, J. 1982. The Biology of Mysids and Euphausiids, The University of Chicago Press.

Mauchline, J. 1998. The biology of calanoid copepods, Academic Press.

Mauchline, J., and L. R. Fisher. 1969. The Biology of Euphausiids, Academic Press.

Mcdermott, J. J., and P. Roe. 1985. Food, Feeding Behavior and Feeding Ecology of Nemerteans. Am Zool **25**: 113–125. doi:[10.1093/icb/25.1.113](https://doi.org/10.1093/icb/25.1.113)

Mills, C. 2017. Phylum Ctenophora: list of all valid scientific names.

Moazzam, M., and N. Moazzam. 2019. Occurrence and Abundance of Commensal Polychaete Hipponoe Gaudichaudi (Family: Amphinomidae): An associate of Goose Barnacle Lepas (Anatifa) Anserifera along Pakistan Coast. International Journal of Biotechnology **16**: 755–759.

Moreno-Alcántara, M. 2019. Atlantidae (Pterotracheoidea) of the northeast Pacific. Zoosymposia **13**: 139–146. doi:[10.11646/zoosymposia.13.1.14](https://doi.org/10.11646/zoosymposia.13.1.14)

Nishiyama, E. Y., E. M. Araujo, and O. M. P. Oliveira. 2016a. Species of Lensia (Cnidaria: Hydrozoa: Siphonophorae) from southeastern Brazilian waters. Zoologia (Curitiba) **33**: e20160030. doi:[10.1590/s1984-4689zool-20160030](https://doi.org/10.1590/s1984-4689zool-20160030)

Nishiyama, E. Y., G. C. Ribeiro, and O. M. P. Oliveira. 2016b. Phylogenetic analysis of the genus Lensia (Cnidaria, Hydrozoa, Siphonophora), based on the species morphology. Zootaxa **4132**: 493. doi:[10.11646/zootaxa.4132.4.2](https://doi.org/10.11646/zootaxa.4132.4.2)

Norenburg, J. L., and S. A. Stricker. 2001. Phylum Nemertea, p. 163–177. *In* Atlas of Marine Invertebrate Larvae.

Ohman, M. D., and J. A. Runge. 1994. Sustained fecundity when phytoplankton resources are in short supply: Omnivory by Calanus finmarchicus in the Gulf of St. Lawrence. Limnology and Oceanography **39**: 21–36. doi:[10.4319/lo.1994.39.1.0021](https://doi.org/10.4319/lo.1994.39.1.0021)

Omori, M. 1975. The Biology of Pelagic Shrimps in the Ocean, p. 233–324. *In* F.S. Russell and M. Yonge [eds.], Advances in Marine Biology. Academic Press.

Pelagic Invertebrates Collection. 2022. Zooplankton of the San Diego Region. Zooplankton Guide.

Pomerleau, C., A. R. Sastri, and B. E. Beisner. 2015. Evaluation of functional trait diversity for marine zooplankton communities in the Northeast subarctic Pacific Ocean. J Plankton Res **37**: 712–726. doi:[10.1093/plankt/fbv045](https://doi.org/10.1093/plankt/fbv045)

Raisuddin, S., K. W. H. Kwok, K. M. Y. Leung, D. Schlenk, and J.-S. Lee. 2007. The copepod Tigriopus: A promising marine model organism for ecotoxicology and environmental genomics. Aquatic Toxicology **83**: 161–173. doi:[10.1016/j.aquatox.2007.04.005](https://doi.org/10.1016/j.aquatox.2007.04.005)

Rampal, J. 2017. Euthecosomata (Mollusca, Gastropoda, Thecosomata). Taxonomic review. 098475. doi:[10.1101/098475](https://doi.org/10.1101/098475)

Razouls, C., N. Desreumaux, J. Kouwenberg, and F. de Bovee. 2005. Biodiversity of Marine Planktonic Copepods (morphology, geographical distribution and biological data).

Reish, D. J. 1961. A New Species of Micronereis (Annelida, Polychaeta) from the Marshall Islands! Pacific Science **15**: 5.

Rouse, G., F. Pleijel, and E. Tilic. 2022. Annelida, Oxford University Press.

Russell, F. S. 1970. The Medusae of the British Isles volume II: Pelagic Scyphozoa, with a supplement to the first volume of Hydromedusae, Cambridge University Press.

Schiariti, A., A. C. Morandini, G. Jarms, R. von G. Paes, S. Franke, and H. Mianzan. 2014. Asexual reproduction strategies and blooming potential in Scyphozoa. Marine Ecology Progress Series **510**: 241–253. doi:[10.3354/meps10798](https://doi.org/10.3354/meps10798)

Schick, J. M. 1991. A Functional Biology of Sea Anemones, 1st ed. Springer Dordrecht.

Schmidt, K. 2010. Food and Feeding in Northern Krill (Meganyctiphanes norvegica Sars), p. 127–171. *In* G.A. Tarling [ed.], Advances in Marine Biology. Academic Press.

Schuchert, P. 2022. World Hydrozoa Database.doi:[10.14284/357](https://doi.org/10.14284/357)

Sheader, M. 1986. *Primno evansi* sp. nov. (Amphipoda: Hyperiidea) from the eastern North Atlantic. Journal of Natural History **20**: 975–980. doi:[10.1080/00222938600770711](https://doi.org/10.1080/00222938600770711)

Simmons, K. 2009. Observations of the egg cases from the holopelagic polychaete family: Tomopteridae. Monterey Bay Aquarium Research Institute.

van der Spoel, S., L. Newman, and K. W. Estep. 1997. Pelagic molluscs of the world,.

Stenvers, V. I., B. C. Gonzalez, F. E. Goetz, J. M. Hemmi, A.-L. Jessop, C. Lin, H.-J. T. Hoving, and K. J. Osborn. 2021. Extraordinary eyes reveal hidden diversity within the holopelagic genus Paraphronima (Amphipoda: Hyperiidea). Deep Sea Research Part I: Oceanographic Research Papers **177**: 103610. doi:[10.1016/j.dsr.2021.103610](https://doi.org/10.1016/j.dsr.2021.103610)

Stevens, B. A., and F. A. Chace. 1965. The Mesopelagic Caridean Shrimp Notostomus japonicus Bate in the Northeastern Pacific. Crustaceana **8**: 277–284.

Taghon, G. L., and R. R. Greene. 1992. Utilization of deposited and suspended particulate matter by benthic “interface” feeders. Limnology and Oceanography **37**: 1370–1391. doi:[10.4319/lo.1992.37.7.1370](https://doi.org/10.4319/lo.1992.37.7.1370)

Takahashi, K. 2004. Feeding ecology of mysids in freshwater and coastal marine habitats: A review. Bulletin of the Plankton Society of Japan **51**: 46–72.

Tesch, J. J. 1908. Systematic monograph of the Atlantidae (Heteropoda) with enumeration of the species in the Leyden museum. Notes from the Leyden Museum **30**: 1–30.

Thomson, C. W., J. Murray, G. S. Nares, and F. T. Thomson. 1889. Report on the Scientific Results of the Voyage of HMS Challenger During the Years 1873-76 Under the Command of Captain George S. Nares... and the Late Captain Frank Tourle Thomson, RN, HM Stationery Office.

Tryon, G. W. 1871. American journal of conchology, Conchological Section of the Academy of Natural Sciences of Philadelphia.

Uhlir, C., M. Schwentner, K. Meland, and others. 2021. Adding pieces to the puzzle: insights into diversity and distribution patterns of Cumacea (Crustacea: Peracarida) from the deep North Atlantic to the Arctic Ocean. PeerJ **9**: e12379. doi:[10.7717/peerj.12379](https://doi.org/10.7717/peerj.12379)

van Couwelaar. 2003. Zooplankton and Micronekton of the North Sea.

Vanhöffen, E. 1902. Die acraspeden Medusen der deutschen Tiefsee-Expedition 1898-1899. Mit Tafel I-VIII, G. Fischer.

Venkatesan, V., and K. S. Mohamed. 2015. 09 Cephalopod classification and Taxonomy. 5.

Vinogradov, M. Y., A. F. Volkov, and T. N. Semyonova. 1982. Amphipody-hyperiidy Mirovogo okeana (Hyperiid Amphipods of the World Ocean), Leningrad: Nauka.

Wall-Palmer, D. 2017. Shelled Heteropod Identification Portal | Atlantidae. ship.

Wicksten, M. K. 2012. Decapod Crustacea of the Californian and Oregonian Zoogeographic Provinces. 307.

Wilson, W. H. 1991. Sexual Reproductive Modes in Polychaetes: Classification and Diversity. BULLETIN OF MARINE SCIENCE **48**: 17.

Wrobel, D., and C. E. Mills. 1998. Pacific Coast Pelagic Invertebrates: A Guide to the Common Gelatinous Animals, Monterey Bay Aquarium.

Yamada, Y., and T. Ikeda. 2003. Metabolism and chemical composition of four pelagic amphipods in the Oyashio region, western subarctic Pacific Ocean. Marine Ecology Progress Series **253**: 233–241.

Yamazaki, T., and T. Kuwahara. 2017. A new species of *Clione* distinguished from sympatric *C. limacina* (Gastropoda: Gymnosomata) in the southern Okhotsk Sea, Japan, with remarks on the taxonomy of the genus. J. Mollus. Stud. **83**: 19–26. doi:[10.1093/mollus/eyw032](https://doi.org/10.1093/mollus/eyw032)

Zeidler, W. 2003. A review of the hyperiidean amphipod family Cystisomatidae Willemöes-Suhm, 1875 (Crustacea: Amphipoda: Hyperiidea). Zootaxa **141**: 1. doi:[10.11646/zootaxa.141.1.1](https://doi.org/10.11646/zootaxa.141.1.1)

Zeldis, J., M. R. James, J. Grieve, and L. Richards. 2002. Omnivory by copepods in the New Zealand Subtropical Frontal Zone. Journal of Plankton Research **24**: 9–23. doi:[10.1093/plankt/24.1.9](https://doi.org/10.1093/plankt/24.1.9)
